# Thyroid dysfunction and breast cancer risk among women in the UK Biobank cohort

**DOI:** 10.1002/cam4.3978

**Published:** 2021-05-26

**Authors:** Thi‐Van‐Trinh Tran, Camille Maringe, Sara Benitez Majano, Bernard Rachet, Marie‐Christine Boutron‐Ruault, Neige Journy

**Affiliations:** ^1^ Epidemiology of radiation Group Center for Research in Epidemiology and Population Health INSERM U1018 Paris Sud‐Paris Saclay University Villejuif France; ^2^ Inequalities in Cancer Outcomes Network Department of Non‐Communicable Disease Epidemiology London School of Hygiene and Tropical Medicine London UK; ^3^ Health across Generations Team Center for Research in Epidemiology and Population Health INSERM U1018 Paris Sud‐Paris Saclay University Villejuif France

**Keywords:** breast cancer, cohort study, hyperthyroidism, hypothyroidism, incidence

## Abstract

This study aimed to evaluate the association between thyroid dysfunction and breast cancer risk. We included 239,436 females of the UK Biobank cohort. Information on thyroid dysfunction, personal and family medical history, medications, reproductive factors, lifestyle, and socioeconomic characteristics was retrieved from baseline self‐reported data and hospital inpatient databases. Breast cancer diagnoses were identified through population‐based registries. We computed Cox models to estimate hazard ratios (HRs) of breast cancer incidence for thyroid dysfunction diagnosis and treatments, and examined potential confounding and effect modification by comorbidities and breast cancer risk factors. In our study, 3,227 (1.3%) and 20,762 (8.7%) women had hyper‐ and hypothyroidism prior to the baseline. During a median follow‐up of 7.1 years, 5,326 (2.2%) women developed breast cancer. Compared to no thyroid dysfunction, there was no association between hypothyroidism and breast cancer risk overall (HR = 0.93, 95% confidence interval (CI): 0.84–1.02, 442 cases), but we found a decreased risk more than 10 years after hypothyroidism diagnosis (HR=0.85, 95%CI 0.74–0.97, 226 cases). There was no association with hyperthyroidism overall (HR=1.08, 95%CI 0.86–1.35, 79 cases) but breast cancer risk was elevated among women with treated hyperthyroidism (HR=1.38, 95%CI: 1.03–1.86, 44 cases) or aged 60 years or more at hyperthyroidism diagnosis (HR=1.74, 95%CI: 1.01–3.00, 113 cases), and 5–10 years after hyperthyroidism diagnosis (HR=1.58, 95%CI: 1.06–2.33, 25 cases). In conclusion, breast cancer risk was reduced long after hypothyroidism diagnosis, but increased among women with treated hyperthyroidism. Future studies are needed to determine whether the higher breast cancer risk observed among treated hyperthyroidism could be explained by hyperthyroidism severity, type of treatment or aetiology.

## Graphical abstract

In this study, we included 239,436 women in the general population‐based UK Biobank cohort to investigate the association between thyroid dysfunction and breast cancer risk. Our study suggested there was no association between hyper‐ and hypothyroidism in overall and breast cancer risk. However, among treated hyperthyroidism, we found a higher breast cancer risk, which could be explained by hyperthyroidism severity or aetiology.

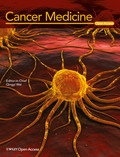

## 1 INTRODUCTION

Breast cancer is the most frequent female neoplasm, with 522,500 new cases diagnosed in Europe in 2018.^1^ It has a peak incidence at the age of 50–70 years––a feature shared by thyroid dysfunction, one of the most common endocrine disorders in females. Experimental data showed that thyroxine (T4) and triiodothyronine (T3) have proliferative and anti‐apoptotic effects on breast cancer tumour cells by regulating gene expression and stimulating oestrogen‐like effects,^2^, ^3^ indicating an association between thyroid dysfunction and breast cancer risk.

However, epidemiological studies have provided inconsistent findings.^4^ Several studies reported higher blood levels of thyroid stimulating hormone (TSH), a biomarker of hypothyroidism, to be associated with a reduced breast cancer risk,^5^, ^6^ while others reported no association.^7^, ^8^, ^9^ In a meta‐analysis of observational studies published up to 2019, no statistically significant association between hypothyroidism and breast cancer risk was found,^4^ but two more recent studies reported a reduced breast cancer risk associated with hypothyroidism.^5^, ^10^ Conversely, some studies,^11^, ^12^, ^13^, ^14^ but not all,^8^, ^10^ showed a higher breast cancer risk among hyperthyroid women compared to those without thyroid dysfunction, which was supported by results from the meta‐analysis ^4^ and a Mendelian randomization study.^5^ This could be, at least partly, due to hyperthyroidism treatments, since radioactive iodine (RAI) therapy has been associated with an increased breast cancer risk,^13^, ^15^ but few studies had this information.

In this study, we aimed to estimate the association between hyper‐ and hypothyroidism (from self‐report and medical records) and breast cancer risk among pre‐ and postmenopausal women, and investigated possible confounding or modifying effects of thyroid dysfunction treatment, comorbidities, and breast cancer risk factors, using data from the 2006–2010 cohort of the population‐based UK Biobank (UKB) cohort.

## 2 MATERIALS AND METHODS

### 2.1 Study population and data sources

From 2006 to 2010, the UKB cohort enrolled 273,375 women from the general population. Participants were volunteers aged from 39 to 71 years, and residing in England, Wales, or Scotland, who gave their written informed consents.^16^ Detailed information on personal and family medical history, medications, reproductive and lifestyle factors, and socioeconomic and demographic characteristics was collected through a self‐reported questionnaire, an interview with a trained nurse, and physical measurements at baseline. The cohort was linked to regional, population‐based registries to collect hospital inpatient diagnostics and procedures (data availability starting between 1981 and 1998 depending on the region), cancer registration (since 1957 to 1971 depending on the region), and death registration data (since 2006). Since the registries did not cover the participants’ lives earlier than their availability date, we used those data sources for follow‐up purposes, and both self‐reported and registry‐based data for baseline information (e.g. pre‐existing cancer at baseline).

**FIGURE 1:**
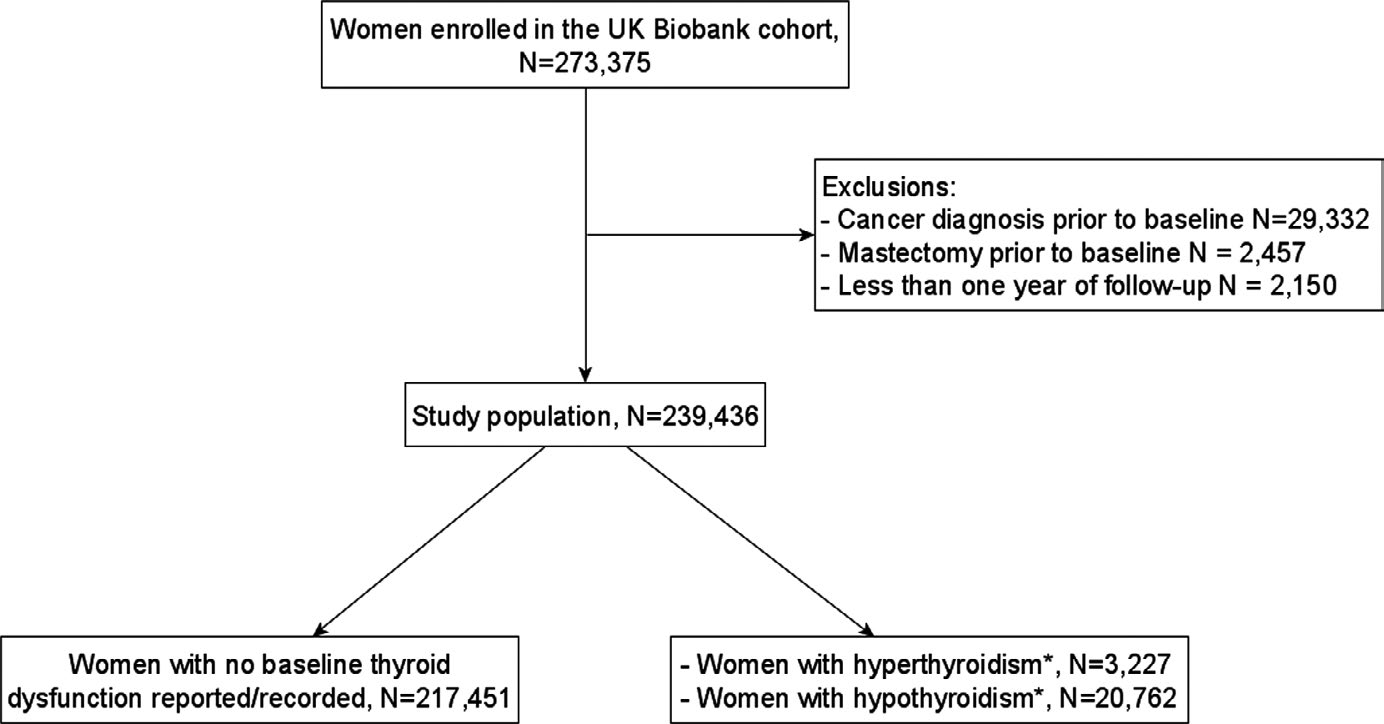
Flowchart of the study population. * Women with both hyper‐ and hypothyroidism reported/recorded (n = 2,004) contributed to both columns of hyper‐ and hypothyroidism

We included participants without cancer diagnosis of any type (except non‐melanoma skin cancer) that was self‐reported or recorded in cancer registries prior to baseline, i.e. the first visit at a UKB centre for study enrollment. We excluded women who underwent a mastectomy prior to baseline, or had less than one year of follow‐up. After exclusions, our study population included 239,436 women (Figure 1). Follow‐up time started at baseline and ended at the date of any cancer diagnosis (except non‐melanoma skin cancer), mastectomy, death, lost‐to‐follow‐up, or 31 March 2016, whichever occurred first.

### 2.2 Exposure

In the primary analyses, we used information on baseline thyroid dysfunction diagnosis (hyperthyroidism, hypothyroidism, no thyroid dysfunction reported/recorded) and treatments [hyperthyroidism: antithyroid drugs (ATDs) (carbimazole, propylthiouracil), RAI, thyroidectomy; hypothyroidism: thyroid hormones (liothyronine, thyroxine)] that was self‐reported during the baseline interview or recorded (at least once) in a hospital inpatient database prior to baseline (Table 1, Appendix 1). Hyperthyroidism and hypothyroidism were assessed separately.

**TABLE 1:** Sources of information and coding used to define thyroid dysfunction diagnosis and treatments

		Self‐reported data at baseline	Hospital inpatient databases			
			ICD−9	ICD−10	OPCS−3	OPCS−4
Thyroid dysfunction diagnosis						
Hyperthyroidism		Graves’ disease Hyperthyroidism Thyroid radioablation therapy Regular use of propilthiouracil or carbimazole at baseline	242	E05	988	X655
Hypothyroidism		Hypothyroidism Regular use of liothyronine or thyroxine at baseline	244, 2452	E02, E032‐E039, E063, E089	NA	NA
Thyroid dysfunction treatment						
Hyperthyroidism treatment	Radioactive iodine^a^	Thyroid radioablation therapy	NA	NA	988	X655
	Surgery^a^, ^b^	Thyroidectomy	NA	NA	070, 071, 072	B08
	Antithyroid drugs only^c^	Regular use of propilthiouracil or carbimazole at baseline	NA	NA		
Hypothyroidism treatment	Thyroid hormones	Regular use of liothyronine or thyroxine at baseline	NA	NA		

Abbreviations: ICD, International classification of diseases; NA, not applicableOPCS, OPCS Classification of Interventions and Procedures.

^a^ Only the first definitive hyperthyroidism treatment was considered, e.g. if radioactive iodine occurred before surgery, the treatment modality was coded as “radioactive iodine”.

^b^ Only procedures performed after a diagnosis of hyperthyroidism were considered.

^c^ If patients were treated with both antithyroid drugs and radioactive iodine/surgery, the treatment modality was coded as “radioactive iodine” or”surgery”, whichever occurred first.

We investigated the following exposure variables: ever diagnosis of hyper‐/hypothyroidism, thyroid dysfunction treatment modalities, time since diagnosis, time since treatment onset, age at diagnosis and calendar year of diagnosis.

### 2.3 Outcome

Breast cancer cases were defined as diagnoses of invasive (n=4,452) or in situ cancers (n=874) recorded in the cancer registries (ICD‐10: C50 or D05, ICD‐9: 174 or 2330). We considered only first cancer occurrences. Women diagnosed with cancer of any type during follow‐up (except non‐melanoma skin cancer) were censored on diagnosis date.

### 2.4 Potential confounders or effect modifiers

We considered comorbidities and breast cancer risk factors, and healthcare‐related factors at baseline as potential confounders or effect modifiers (Appendix 2).

The comorbidities of interest were overweight/obesity, hypertension, diabetes, depression, and autoimmune conditions. Type 1 and 2 diabetes were identified using a modified version of a published algorithm that was developed using the UKB data and validated against external primary and secondary care databases (Appendix 3).^17^ Since thyroid dysfunction aetiology was not systematically recorded in the UKB and various autoimmune conditions can occur among patients with thyroid autoimmune diseases such as Graves’ or Hashimoto's disease,^18^ we investigated a potential modifying effect by autoimmune conditions as a proxy for the autoimmune aetiology of thyroid dysfunction. We used a variable including any autoimmune condition other than autoimmune thyroid diseases at baseline (Appendix 4).^19^, ^20^, ^21^

We considered well‐established breast cancer risk factors as potential confounders or effect modifiers: menopausal status, age at menarche, parity, age at first birth, family history of breast cancer, use of menopausal hormone therapy (MHT), use of oral contraception, and level of physical activities. Baseline age at menopause was defined as age at bilateral oophorectomy or reported menopause whichever occurred first. If unknown, it was defined in order of priority as age at MHT initiation, or 51 years otherwise. The age threshold corresponds to the median value of age at menopause in the study population.

Other factors suggested to be possibly associated with breast cancer risk in the literature were considered as potential confounders: race, alcohol consumption, and smoking status. Townsend deprivation score of residence, educational attainment, occupation, and adherence to breast and cervical cancer screening programs, which might reflect different levels of health care access and cancer surveillance, were also analysed as potential confounders.

For all the above‐mentioned factors, missing data were infrequent (<5%, except age at menopause: 9.3%), and handled either by defining an “unknown” category (for categorical variables) or imputing the median value in the study population (for continuous variables).

### 2.5 Statistical analyses

We used Cox proportional hazards models to compute hazards ratios (HRs) and 95% confidence intervals (CIs) of breast cancer incidence according to thyroid dysfunction diagnosis and treatments. Time since baseline (i.e. UKB inclusion) was considered as the time scale. Models were adjusted for age at baseline, menopausal status, family history of breast cancer, parity and age at first birth, and level of physical activity. Proportional hazards assumptions were graphically evaluated based on plots of scaled Schoenfeld residuals against time, and tested by introducing an interaction term between thyroid dysfunction and follow‐up time. No evidence of non‐proportionality was found.

Potential confounding effect was assessed by evaluating the age‐adjusted associations with thyroid dysfunction, and changes in adjusted HRs for breast cancer risk exceeding 10%.^22^ Effect modification was evaluated by testing the statistical significance of an interaction term between thyroid dysfunction and the studied covariate [likelihood‐ratio χ² tests for heterogeneity (categorical variables) and linear trend (continuous variables)]. When statistically significant multiplicative interactions were detected, we reported results for both additive and multiplicative interactions.^23^

Several sensitivity analyses were conducted. While hyperthyroid patients are usually treated, the proportion of hyperthyroid women without information on treatment was too high to be considered as untreated individuals. Since ATDs are used as the first‐line treatment for Graves’ disease (the most common cause of hyperthyroidism) in the UK ^24^, ^25^ and was more likely to be missed when retrieving information compared to surgery and RAI in the UKB, we hypothesized that women with no information on treatments were treated with ATDs, and conducted sensitivity analyses while combining them and women treated with ATDs. Thyroidectomy can be used to treat other thyroid diseases and thyroid dysfunction could be a transient condition before other thyroid disorders, therefore, we excluded women with other baseline thyroid problems, e.g. thyroiditis, and non‐toxic goitre. To minimize misclassification, we conducted an analysis stratified by the order of thyroid dysfunction occurrence and excluded women who had hypothyroidism reported/recorded before hyperthyroidism or who had hyper‐ and hypothyroidism reported/recorded with unknown sequential order of occurrence. We also analysed separately thyroid dysfunction diagnoses and treatments recorded in the hospital databases only (likely reflecting the most severe conditions), and self‐reported data only to assess the impact of the data sources on the results. We added information on new thyroid dysfunction diagnoses and hyperthyroidism treatment identified in the hospital databases during follow‐up, by considering exposure as a time‐dependent variable. We evaluated the association between thyroid dysfunction and invasive breast cancer risk only. We also computed cause‐specific hazard models ^26^ to consider death and non‐breast cancer incidence as competing risks. Lastly, we did a sensitivity analysis by excluding women with missing data in covariates.

## 3 RESULTS

### 3.1 Population description

The prevalence of hyper‐ and hypothyroidism at baseline was 1.3% and 8.7%, respectively. Compared to women with no thyroid dysfunction, hyper‐ and hypothyroid women were likely to be older, postmenopausal, MHT and oral contraception ever user, obese/overweight, to have had a child at an earlier age, to have a lower level of physical activity, and more comorbidities at baseline (Table 2, Table S1). During a median follow‐up time of 7.1 years, 5326 (2.2%) women were diagnosed with breast cancer.

**TABLE 2:** Baseline characteristics of the study population (n = 239,436)

	No thyroid dysfunction reported (n = 217,451)	Hyperthyroidism (n = 3,227)^a^		Hypothyroidism (n = 20,762)^a^	
			*p*‐value^b^		*p* ‐value^b^
Person‐years of follow‐up, median (IQR)	7.1 (6.4, 7.8)	7.1 (6.4, 7.8)	0.377	7.0 (6.4, 7.8)	< 0.001
Age at baseline, Mean (SD)	56.4 ± 8.1	58.0 ± 7.6	<0.001	58.9 ± 7.3	<0.001
Menopause status, n (%)			<0.001		<0.001
Still had periods	60,047 (27.6)	612 (19.0)		3,189 (15.4)	
Had menopause before the age of 51	106,860 (49.1)	1,764 (54.7)		12,105 (58.3)	
Had menopause after the age of 51	50,544 (23.2)	851 (26.4)		5,468 (26.3)	
Age at menopause^c^, Mean (SD)	49.3 ± 5.1	49.2 ± 5.4	0.918	49.0 ± 5.5	< 0.001
Age at menarche, Mean (SD)	13.0 ± 1.6	12.9 ± 1.6	0.213	12.9 ± 1.6	<0.001
Family history of breast cancer, n (%)	22,951 (10.6)	309 (9.6)	0.077	2,113 (10.2)	0.093
Ever use of menopausal hormone therapy^c^, n (%)			<0.001		<0.001
No	77,660 (49.3)	1,193 (45.6)		7,473 (42.5)	
Yes, for less than 5 years	27,624 (17.5)	442 (16.9)		3,206 (18.2)	
Yes, for more than 5 years	41,604 (26.4)	764 (29.2)		5,384 (30.6)	
Yes, unknown duration	9,723 (6.2)	206 (7.9)		1,432 (8.1)	
Unknown	793 (0.5)	10 (0.4)		78 (0.4)	
Ever use of oral contraception, n (%)			<0.001		<0.001
No	39,784 (18.3)	723 (22.4)		4,722 (22.7)	
Yes, for less than 10 years	78,956 (36.3)	1,216 (37.7)		7,855 (37.8)	
Yes, for more than 10 years	78,019 (35.9)	972 (30.1)		6,070 (29.2)	
Yes, unknown duration	20,338 (9.4)	308 (9.5)		2,078 (10.0)	
Unknown	354 (0.2)	8 (0.2)		37 (0.2)	
Parity and age at first birth, n (%)			<0.001		<0.001
No live birth	41,026 (18.9)	567 (17.6)		3,336 (16.1)	
≥ one child, <30 years old at birth	135,291 (62.2)	2,134 (66.1)		14,340 (69.1)	
≥ one child, ≥30 years old at birth	40,071 (18.4)	516 (16.0)		3,007 (14.5)	
Unknown	1,063 (0.5)	10 (0.3)		79 (0.4)	
Corpulence, n (%)			0.024		<0.001
Obesity/Overweight, BMI ≥25 kg/m²	128,257 (59.0)	1,974 (61.2)		14,564 (70.1)	
Normal weight/Underweight, BMI <25 kg/m^2^	88,047 (40.5)	1,239 (38.4)		6,107 (29.4)	
Unknown	1,147 (0.5)	14 (0.4)		91 (0.4)	
Comorbidities, n (%)			<0.001		<0.001
Type 2 diabetes	6,534 (3.0)	166 (5.1)		1,203 (5.8)	
Hypertension	49,848 (22.9)	1,006 (31.2)		6,579 (31.7)	
Depression	15,145 (7.0)	263 (8.1)		2,027 (9.8)	
Autoimmune diseases	20,263 (9.3)	450 (13.9)		2,851 (13.7)	
Levels of physical activities, n (%)			0.002		<0.001
Low	68,804 (31.6)	1,106 (34.3)		7,438 (35.8)	
Moderate	77,862 (35.8)	1,146 (35.5)		7,084 (34.1)	
High	70,785 (32.6)	975 (30.2)		6,240 (30.1)	

Abbreviation: BMI, Body‐mass index

^a^ Women with both hyper‐ and hypothyroidism reported/recorded (n=2,004) contributed to both columns of hyper‐ and hypothyroidism.

^b^ *p*‐value of t‐test, Mann‐Whitney U test and χ^2^ test, where appropriate.

^c^ Postmenopausal women only.

### 3.2 Hyperthyroidism

We found no statistically significant association between breast cancer risk and hyperthyroidism in overall (Table 3), but an increased risk at 5–10 years after hyperthyroidism diagnosis (HR=2.38, 95% CI 1.19–4.76), among women who were diagnosed with hyperthyroidism after the age of 60 years (HR=1.74, 95% CI 1.01–3.00), or among women who were treated for hyperthyroidism (HR=1.38, 95% CI 1.03–1.86). Stratification by treatment status showed that the increase of risk among women who were diagnosed with hyperthyroidism for 5–10 years or at the age of 60 years or more, only concerned treated individuals, while there was no association among women with no information on treatment (Table A3). The results did not substantially differ in sensitivity analyses (Tables S4, S6, Figure S1).

**TABLE 3:** Hazard ratios of breast cancer incidence associated to thyroid dysfunction diagnosis and treatment versus no thyroid dysfunction at baseline

Characteristics	Hyperthyroidism			Hypothyroidism		
	No. of breast cancer cases/Person‐years	HR	95%CI	No. of breast cancer cases/Person‐years	HR	95%CI
No thyroid dysfunction (reference)	4,854/1,518,670	**1**	**—**	4,854/1,518,670	1	—
Overall	79/22,520.6	1.08	0.86, 1.35	442/144,213.1	0.93	0.84, 1.02
Age at diagnosis						
Before 40 years old	20/8,436.4	0.75	0.48, 1.16	50/23,615.7	**0.71**	**0.54, 0.94**
Between 40–60 years old	45/11,725	1.18	0.88, 1.58	271/87,945.4	0.94	0.83, 1.06
After 60 years old	13/2,007.2	**1.74**	**1.01, 3.00**	70/16,728.3	1.11	0.88, 1.41
Unknown age at diagnosis	1/352	0.92	0.13, 6.56	51/15,923.6	0.97	0.73, 1.27
P‐trend^a^			*0.145*			*0.452*
Time since diagnosis						
Less than 5 years ago	4/2,333.6	0.70	0.26, 1.87	34/15,154.9	0.91	0.65, 1.27
Between 5–10 years ago	25/4,947.4	**1.58**	**1.06, 2.33**	131/37,024.4	1.08	0.91, 1.29
More than 10 years ago	49/14,887.6	0.97	0.73, 1.29	226/76,110.1	**0.85**	**0.74, 0.97**
Unknown time at diagnosis	1/352	0.91	0.13, 6.47	51/15,923.6	0.99	0.75, 1.30
P‐trend^a^			*0.124*			*0.872*
Calendar year at diagnosis						
Before 1990	26/6,910.3	1.09	0.74, 1.61	47/17,558.6	0.79	0.59, 1.05
1990–2000	14/5,946.9	0.74	0.44, 1.24	120/40,954.8	0.88	0.73, 1.05
After 2000	38/9,317.3	1.29	0.94, 1.77	224/69,814.3	0.99	0.86, 1.13
Unknown time at diagnosis	1/346.2	0.93	0.13, 6.60	51/15,885.4	0.96	0.73, 1.27
P‐trend^a^			*0.366*			*0.352*
Treatment status						
Without information on treatment (1)	35/12,816.1	0.84	0.60, 1.17	22/4,831.6	1.39	0.91, 2.11
With information on treatment	44/9,704.5	**1.38**	**1.03, 1.86**	420/139,381.5	0.91	0.83, 1.01
Types of hyperthyroidism treatment						
Antithyroid medications (2)	9/1,978	1.46	0.76, 2.81			
RAI (3)	11/2,697.4	1.23	0.68, 2.23			
Surgery (4)	24/5,029.1	1.44	0.96, 2.15			
(1) or (2)	44/14,794.1	0.92	0.68, 1.24			
(3) or (4)	35/19,822.2	1.37	0.98,1.91			
Time since hyperthyroidism treatment						
Less than 5 years ago	1/435.2	0.97	0.14, 6.90			
Between 5–10 years ago	8/1,068.6	**2.38**	**1.19, 4.76**			
More than 10 years ago	26/6,192.9	1.24	0.84, 1.82			
Unknown time at diagnosis	9/2,007.8	1.43	0.74, 2.74			
P‐trend^a^		*0.044*				

Note

HRs are adjusted for age at baseline (continuous), family history of breast cancer (yes/no), parity and number of live birth (No live birth/≥ one child, <30 years old at birth/≥ one child, ≥30 years old at birth/Unknown), menopausal status (premenopause/postmenopause before the age of 51/postmenopause after the age of 51), physical activities (Low/Moderate/High).

Abbreviations: HR, Hazard Ratio, CI, Confidence Interval, RAI, Radioactive iodine therapy.

^a^ p‐trend was calculated after excluding hyperthyroidism/hypothyroidism with unknown time at diagnosis/treatment.

For treated hyperthyroidism, there was a higher breast cancer risk among women menopaused at ages >51 years (HR=2.07, 95% CI 1.33–3.22) compared to women who had earlier menopause (HR=1.18, 95% CI 0.76–1.83) or were premenopausal at baseline (HR=0.79, 95% CI 0.30–2.11) (p‐heterogeneity=0.09) (Table 4). We found no confounding or modifying effect by comorbidities, and breast cancer risk factors, except hypertension based on very few cases (Figure S2).

**TABLE 4:** Breast cancer risk associated with treated hyperthyroidism according to baseline menopausal status and age at menopause

Menopausal status and age at menopause	N with/without breast cancer	HR (95% CI)	HR (95% CI) within strata of menopausal status and age at menopause
Premenopause			
No thyroid dysfunction	1,194/58,853	1.19 (1.07–1.31), *p* = 0.001	1.00
Treated hyperthyroidism	4/248	0.91 (0.34–2.44), *p* = 0.856	0.77 (0.29–2.05), *p* = 0.601
Having menopause before the age of 51			
No thyroid dysfunction	2,341/104,519	1.00	1.00
Treated hyperthyroidism	20/743	1.19 (0.76–1.84), *p* = 0.443	1.19 (0.76–1.84), *p* = 0.443
Having menopause after the age of 51			
No thyroid dysfunction	1,319/49,225	1.16 (1.08–1.24), *p* < 0.001	1.00
Treated hyperthyroidism	20/358	2.39 (1.54–3.71), *p* < 0.001	2.07 (1.33–3.22), *p* = 0.001

Note

Measure of effect modification of premenopause on additive scale: Treated hyperthyroidism: RERI (95% CI)  = −0.46 (−1.5–0.58), *p*  =  0.391. Measure of effect modification of having menopause after the age of 51 on additive scale: Treated hyperthyroidism: RERI (95% CI)  = 1.05 (−0.12–2.22), *p*  =  0.08. Measure of effect modification of premenopause on multiplicative scale: Treated hyperthyroidism: ratio of HRs (95% CI)  = 0.65 (0.22–1.9), *p*  =  0.429. Measure of effect modification of having menopause after the age of 51 on multiplicative scale: Treated hyperthyroidism: ratio of HRs (95% CI)  = 1.74 (0.93–3.25), *p*  =  0.081. HRs are adjusted for age at baseline (continuous), family history of breast cancer (yes/no), parity and number of live birth (No live birth/≥ one child, <30 years old at birth/≥ one child, ≥30 years old at birth/Unknown), and physical activities (Low/Moderate/High).

### 3.3 Hypothyroidism

We found no statistically significant association between hypothyroidism and breast cancer risk, overall (HR=0.93, 95% CI 0.84–1.02), or after stratification by calendar year at diagnosis or treatment (Table 3). However, there was a lower risk among women diagnosed with hypothyroidism before the age of 40 years (HR=0.71, 95% CI 0.54–0.94) or diagnosed for hypothyroidism for more than 10 years (HR=0.85, 95% CI 0.74–0.97). The results did not substantially differ in sensitivity analyses (Tables S5, S6 , Figure S1).

We found no confounding or modifying effect by comorbidities, and breast cancer risk factors (Figure S2), except age at menopause. We observed lower risks among premenopausal women at baseline (HR=0.69, 95% CI 0.51–0.93) or women menopaused at ages ≤ 51 years (HR=0.90, 95% CI 0.79–1.02) compared to those with later menopause (HR=1.10, 95% CI 0.93–1.30) (p‐value for heterogeneity: 0.017) (Table 5). The results of analyses on age at menopause did not vary after adjustment for age at menopause (for postmenopausal women) and in further analyses stratified by age at baseline, natural or artificial menopause, age at or time since hypothyroidism diagnosis, occurrence of thyroid dysfunction before or after menopause, or use of MHT or not.

**TABLE 5:** Breast cancer risk associated with hypothyroidism according to baseline menopausal status and age at menopause

Menopausal status and age at menopause	N with/without breast cancer	HR (95% CI)	HR (95% CI) within strata of menopausal status and age at menopause
Premenopause			
No thyroid dysfunction	1194/58853	1.19 (1.08–1.32), *p* = 0.001	1.00
Hypothyroidism	44/3145	0.82 (0.61–1.12), *p* = 0.214	0.69 (0.51–0.93), *p* = 0.016
Having menopause before the age of 51			
No thyroid dysfunction	2341/104519	1.00	1.00
Hypothyroidism	240/11865	0.90 (0.79–1.02), *p* = 0.109	0.90 (0.79–1.02), *p* = 0.109
Having menopause after the age of 51			
No thyroid dysfunction	1319/49225	1.15 (1.08–1.24), *p* < 0.001	1.00
Hypothyroidism	158/5310	1.27 (1.08–1.49), *p* = 0.004	1.10 (0.93–1.30), *p* = 0.261

Note

Measure of effect modification of premenopause on additive scale: Hypothyroidism: RERI (95% CI)  = −0.26 (−0.55–0.02), *p*  =  0.066. Measure of effect modification of having menopause after the age of 51 on additive scale: Hypothyroidism: RERI (95% CI)  = 0.22 (−0.02–0.46), *p*  =  0.073. Measure of effect modification of premenopause on multiplicative scale: Hypothyroidism: ratio of HRs (95% CI)  = 0.77 (0.55–1.07), *p*  =  0.121. Measure of effect modification of having menopause after the age of 51 on multiplicative scale: Hypothyroidism: ratio of HRs (95% CI)  = 1.23 (0.99–1.51), *p*  =  0.06. HRs are adjusted for age at baseline (continuous), family history of breast cancer (yes/no), parity and number of live birth (No live birth/≥ one child, <30 years old at birth/≥ one child, ≥30 years old at birth/Unknown), and physical activities (Low/Moderate/High).

## 4 DISCUSSION

In this study, there was no association between thyroid dysfunction and breast cancer risk overall. However, breast cancer risk varied according to hyperthyroidism treatment status, with a 38% higher breast cancer risk in women treated for hyperthyroidism compared to women with no thyroid dysfunction and no increased risk among hyperthyroid women without information on treatment. The risk was particularly elevated at 5–10 years after hyperthyroidism diagnosis and among women diagnosed for hyperthyroidism at the age of 60 years or more. Women with a history of hypothyroidism for 10 years or more or diagnosed before the age of 40 years were at a lower risk of breast cancer. Menopausal status and age at menopause modified the association of both treated hyper‐ and hypothyroidism.

Accumulated evidence in recent years has not provided a clear understanding of the role of hypothyroidism on breast cancer risk. Some ^5^, ^6^, ^10^ but not all studies ^7^, ^8^, ^9^ have suggested that higher blood levels of TSH and thyroid hormone replacement therapy were associated with a reduced risk of breast cancer. Our findings showed an inverse association between breast cancer risk and hypothyroidism among women diagnosed before 40 years of age or only after 10 years of hypothyroidism, in partial agreement with a previous meta‐analysis and a recent study.^4^, ^10^

In the current study, we reported an increased risk of breast cancer among women with treated hyperthyroidism while the meta‐analysis ^4^ and two nationwide hospital cohort studies ^11^, ^12^ suggested a higher risk with hyperthyroidism in general. Of note, in those studies, hyperthyroidism was mainly ascertained through hospital databases, thus, probably mostly including treated cases. One of the cohort studies ^11^ also had an older population compared to ours, which might partly explain the overall elevated risk with hyperthyroidism. In contrast, a recent cohort study did not find any association between self‐reported hyperthyroidism and breast cancer risk, but information on treatment was limited and only available for medications.^10^

In our study, baseline characteristics did not differ substantially between hyperthyroidism with/without information on treatment (Table S2). Increased risk among hyperthyroid women with information on treatments, but not among those without information might be explained by surveillance bias, types of treatment themselves, or treatment‐related factors. Women with treated hyperthyroidism could possibly have more regular health care consultations. However, the increased risk remained after 10 years of diagnosis and did not change after accounting for health care‐related factors. Thus, surveillance bias was unlikely to be a major explanatory factor. Besides, hyperthyroid patients treated with RAI have been suggested to have a higher breast cancer risk, in relation to the radiation dose received. However, as the possible effect of RAI is modest and observed only after a long latency period,^13^, ^15^ it was unlikely the principal cause of the higher breast cancer risk among treated hyperthyroidism in our study. Moreover, we found consistent risks across different types of treatment, suggesting that breast cancer risk in treated hyperthyroidism was unlikely attributable solely to a specific treatment type.

Treatments are generally not recommended in subclinical hyperthyroidism when overt conditions are often treated as soon as diagnosed.^27^ In the current study, hyperthyroid patients without information on treatment could have subclinical hyperthyroidism, which can be endogenous or exogenous (due to overtreated hypothyroidism), while patients with information on treatment might suffer from overt conditions. A recent large population‐based linked‐record study in the UK found that the majority (74%) of patients with Graves’ disease were treated with ATDs.^25^ Since the recommended length of an ATDs course often lasts no longer than 12–18 months, and in the UKB cohort, only ATDs which were regularly being taken at baseline were recorded, and not before, it is possible that hyperthyroid women without information on treatment were actually treated with ATDs, and we found no association with breast cancer risk among these patients in the sensitivity analysis including all those subjects as treated with ATDs. Nevertheless, we always observed higher breast cancer risks among hyperthyroidism treated with definitive treatments (RAI, surgery), which are preferred among patients with recurrent hyperthyroidism (likely having more severe manifestation ^28^) or hyperthyroidism caused by toxic nodular goiter. Previous studies have suggested that the higher breast cancer risk associated with hyperthyroidism was strongest among patients with toxic nodular goiters.^12^, ^29^ In our study, breast cancer risk did not vary when stratifying by the presence of autoimmune disease. Thus, the aetiology of thyroid dysfunction might not be related to the increased risk.

Most,^6^, ^7^, ^9^, ^30^ but not all,^8^ previous studies found breast cancer risk increased with increasing blood levels of thyroxine (a marker of hyperthyroidism severity). A recent study which included women without thyroid medication found that both abnormal high blood levels of thyroxine and thyroxine in the euthyroid range were associated with higher breast cancer risk, but the risk associated with overt hyperthyroidism was higher than that with subclinical conditions.^6^

Biological mechanisms underlying the association between breast cancer risk and TSH remains unclear, but a number of explanation for thyroid hormones have been explored in vitro and in vivo. T4 and T3 activate MPAK pathways and phosphorylate ER α, inducing cell proliferation.^2^, ^3^, ^31^ T3 can also enhance the effect of oestrogens on breast cell proliferation,^32^ and directly increases aerobic glycolysis, a hallmark of cancer, which is known as Warburg effect.^2^ T4 is known to have anti‐apoptotic properties, which act via the integrin α vβ3, by stimulating gene expression of cancer cell defense.^33^, ^34^ Moreover, excessive or insufficient iodine intake, which plays a key role in thyroid hormone production, could also be a risk factor for breast cancer.^35^ Taken together, current experimental evidence supports a positive association between high levels of thyroid hormones and a higher risk of breast cancer.

In this study, the breast cancer risk estimates for thyroid dysfunction were not affected by a wide range of potential confounders and effect modifiers, except menopausal status and late age at menopause irrespective of other factors. Few studies have investigated a potential effect modification by menopausal status and reproductive factors. A recent study showed a positive association between hyperthyroidism and reproductive risk factors of breast cancer.^12^ In a large cohort of postmenopausal women, the reduced risk of breast cancer associated with hypothyroidism disappeared among women who used MHT for any duration.^10^ Some other studies found evidence of a stronger association with high levels of T4 among postmenopausal women compared to premenopausal ones.^6^, ^7^, ^30^ Among postmenopausal women, the association between T4 and breast cancer risk was also stronger among obese women,^30^ who had higher oestrogen blood concentration than women with normal weight.^36^ Taken together, the current evidence suggested that reproductive factors might modify the breast cancer risk associated with thyroid dysfunction.

Late age at menopause has been confirmed risk factor of breast cancer, which lengthens the cumulative exposure to cycling reproductive hormones among women.^37^ After menopause, endogenous oestrogen is produced dominantly by the peripheral conversion of androgens in adipose tissue,^36^ which is represented by BMI. Almost 40%–50% variation in natural age at menopause has been suggested to be attributable to genetic factors.^38^, ^39^ However, in our study, the association between breast cancer and thyroid dysfunction did not vary according to other genetic‐ and oestrogen‐related factors. Besides, although the proportion of ER + and ER‐ breast cancer has been shown to vary according to age at breast cancer diagnosis among both pre‐ and postmenopausal women,^37^ we observed no substantial difference in the distribution of age at breast cancer diagnosis according to thyroid dysfunction and menopausal status. Thus, the underlying biological mechanisms of the effect modification by menopausal status and age at menopause remain unclear.

The current study has major strengths, including a large population size, a high level of follow‐up completeness and outcome ascertainment through regional registries and hospital databases, and its wide range of available information. The crossover among inpatient data and self‐reported data on personal medical history allowed us to capture a broad range of health conditions. The UKB also includes detailed information on reproductive factors, lifestyle, socioeconomic status and family medical history with low levels of missing data, which helped us to study essential risk factors.

However, several limitations can be flagged. Details on cancer stage, grade, and receptor status were unavailable, and we could not investigate whether the risk estimates varied according to tumour characteristics. Lack of information on laboratory measurements of thyroid hormones, aetiology, and clinical symptoms of thyroid dysfunction prevented us from determining the severity, the exposure window of thyroid dysfunction (as overt conditions are often treated as soon as diagnosed) and disentangling the independent role of severity, and aetiology. We were unable to account for thyroid dysfunction diagnosis/treatments for the whole study population during follow‐up or to study the independent effects of thyroid dysfunction treatments, and evaluate the impact of different treatment‐related factors: RAI dosage, partial versus total thyroidectomy, duration of use and adherence to ATDs prescription. In the hyperthyroidism analyses, given that the higher risks were consistent across different types of treatment, accounting for treatment‐related factors is unlikely to change our risk estimates. However, considering the intertwined relationship between hyperthyroidism aetiology, severity and treatment for further research is needed to confirm our finding. The data on comorbidities were also quite limited with no information on severity, age at onset, and duration of conditions, so it is possible that we did not account for all the possible effects of comorbidities on the association between thyroid dysfunction and breast cancer risk.

In conclusion, our study suggested that higher breast cancer risk among hyperthyroid women could be explained by hyperthyroidism severity or aetiology, while there was a lower risk among women with 10 years or more of hypothyroidism. The association between thyroid dysfunction and breast cancer risk was modified by menopausal status and age at menopause, suggesting that the positive association between increased blood levels of thyroid hormones and breast cancer risk was even stronger with late age at menopause.

## CONFLICT OF INTERESTS

The authors declare no conflict of interest.

## ETHICS APPROVAL AND CONSENT TO PARTICIPATE

This study was performed under generic ethical approval obtained by UK Biobank from the National Health Service National Research Ethics Service (approval letter ref 16/NW/0274, 13 May 2016).

## Supporting information

Supplementary Material

## Data Availability

This work has been conducted using the UK Biobank Resource under Application Number 35032. Bona‐fide researchers can apply to use the UK Biobank dataset by registering and applying at http://www.ukbiobank.ac.uk/register‐apply.
