# Supplementary material for: Thyroid dysfunction and breast cancer risk among women in the UK Biobank cohort
Source: Cancer Med. 2021 May 26;10(13):4604–14. doi: 10.1002/cam4.3978 (PMC8267139; doi:10.1002/cam4.3978)
Supplement: Supplementary file 1 — Supplementary Material [file CAM4-10-4604-s001.docx]

**Thyroid dysfunction and breast cancer risk among women in the UK Biobank cohort**

**Supplementary materials**

[Appendix 1: Definition of treatments for hyperthyroidism 2](#_Toc68104406)

[Appendix 2: Definition of confounding factors and effect modifiers 3](#_Toc68104407)

[Appendix 3: Definition of diabetes 6](#_Toc68104408)

[Appendix 4: List of autoimmune diseases considered in the study 8](#_Toc68104409)

[Appendix 5: Supplementary results 10](#_Toc68104410)

[Supplementary table 1: Baseline characteristics of the study population (n=239,436) 10](#_Toc68104411)

[Supplementary table 2: Baseline characteristics of hyperthyroid women (n=3,227) 12](#_Toc68104412)

[Supplementary table 3: Hazard ratios of breast cancer incidence associated with hyperthyroidism diagnosis versus no thyroid dysfunction at baseline, stratified according to treatment status 17](#_Toc68104413)

[Supplementary table 4: Hyperthyroidism sensitivity analyses 18](#_Toc68104414)

[Supplementary table 5: Hypothyroidism sensitivity analyses 22](#_Toc68104415)

[Supplementary table 6: Results from Cox proportional hazard models for death and other cancer incidence as competing risks 26](#_Toc68104416)

[Supplementary figure 1: Cumulative incidence function of breast cancer accounting for death and other cancer incidence as competing risks 26](#_Toc68104417)

[Supplementary figure 2: Evaluation of effect modification of comorbidities, and breast cancer risk factors in the association between thyroid dysfunction and breast cancer risk. 27](#_Toc68104418)

[References 28](#_Toc68104419)

## Appendix 1: Definition of treatments for hyperthyroidism


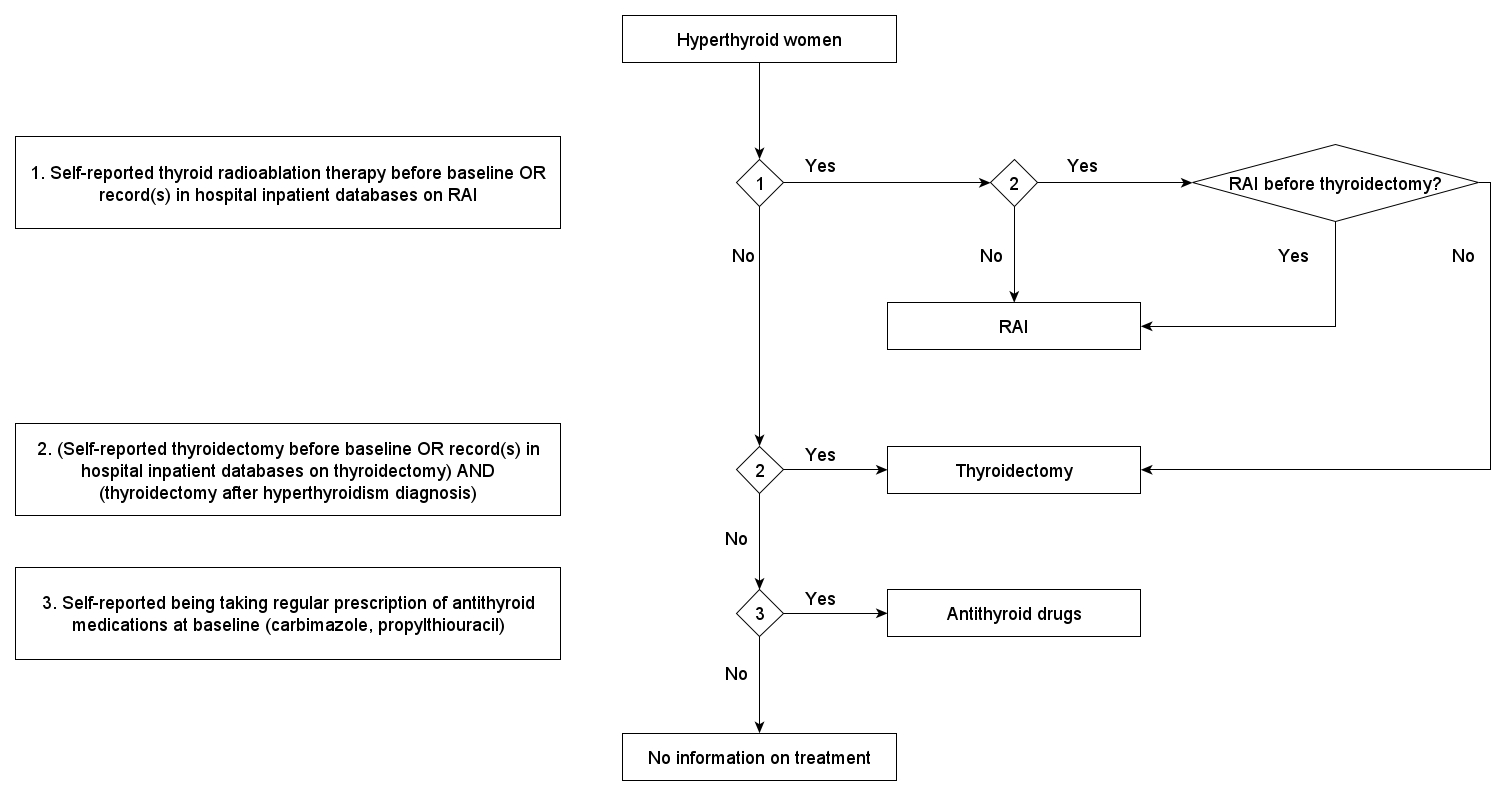


For more information:

- Self-report data on medical conditions, operations, and medications: <https://biobank.ndph.ox.ac.uk/showcase/showcase/docs/Interview.pdf>

- Hospital inpatient databases: <https://biobank.ndph.ox.ac.uk/showcase/showcase/docs/HospitalEpisodeStatistics.pdf>

## Appendix 2: Definition of confounding factors and effect modifiers

| **Risk factors** | **Coding** | **Information sources** | | | | | **Testing for confounding effect** | **Testing for effect modification** |
| --- | --- | --- | --- | --- | --- | --- | --- | --- |
|  |  | **Visit at baseline** | **Hospital inpatient databases^1^** | | | |  |  |
|  |  |  | **ICD9** | **ICD10** | **OPCS3** | **OPCS4** |  |  |
| **Comorbidities** |  |  |  |  |  |  |  |  |
| - Obesity/Overweight | Yes No | BMI (PM): ≥25kg/m^2^ (1) | NA | NA | NA | NA | Yes | Yes |
| -Type 2 diabetes | Yes No | See [appendix](#_Appendix_1:_Definition) 3 | See [appendix](#_Appendix_1:_Definition) 3 | | | | Yes | Yes |
| - Hypertension | Yes No | SR-I | 401 | I10 | NA | NA | Yes | Yes |
| - Depression | Yes No | SR-I | 2962, 2963, 311 | F32, F33 | NA | NA | Yes | Yes |
| - Autoimmune diseases | Yes No | See [appendix](#_Appendix_2:_List) 4 | See [appendix](#_Appendix_2:_List) 4 | | | | Yes | Yes |
| **Hormone-related factors** |  |  |  |  |  |  |  |  |
| - Menopausal status | Premenopause Menopause before 51 years of age Menopause after 51 years of age | - Reporting menopause (periods stopped) (SR-Q)  OR - Reporting use of menopausal hormone therapy (SR-Q)  OR - Undergoing a bilateral oophorectomy (SR-I)  OR - ≥51 years of age at baseline | NA | NA | Bilateral oophorectomy (6812) | Bilateral oophorectomy (Q221) | Yes | Yes |
| - Family history of breast cancer | Yes No | SR-Q | NA | NA | NA | NA | Yes | Yes |
| - Ever use of HRT | Never Yes, for less than 5 years Yes, for more than 5 years Yes, unknown duration Unknown | SR-Q | NA | NA | NA | NA | Yes | Yes |
| - Parity | No live birth One or two live births Three or more live births | SR-Q | NA | NA | NA | NA | Yes | Yes |
| - Age at first birth | No live birth  Before 25 years of age  Between 25-35 years of age  After 35 years of age  Unknown | SR-Q | NA | NA | NA | NA | Yes | Yes |
| - Ever use of oral contraception | Never  Yes, for less than 10 years  Yes, for more than 10 years  Yes, unknown duration Unknown | SR-Q | NA | NA | NA | NA | Yes | Yes |
| - Age at menarche | ≤11 years of age 12-14 years of age  ≥15 years of age  Unknown | SR-Q | NA | NA | NA | NA | Yes | Yes |
| **Other risk factors** |  |  |  |  |  |  |  |  |
| - Physical activity^2^ | Low  Moderate  High | SR-Q | NA | NA | NA | NA | Yes | Yes |
| - Alcohol consumption frequency | Never  Once or twice a week or less  Three times a week or more  Unknown | SR-Q | NA | NA | NA | NA | Yes | No |
| - Smoking status | Never  Former smoker  Current smoker  Unknown | SR-Q | NA | NA | NA | NA | Yes | No |
| - Race | White  Asia Black and Caribbean  Other or unknown | SR-Q | NA | NA | NA | NA | Yes | No |
| **Health care-related factors and socioeconomic characteristics** |  |  |  |  |  |  |  |  |
| - Adherence to mammography guideline | <50 years of age  >50 years of age, >3 years ago  >50 years of age, in the last 3 years  >50 years of age, never  >50 years of age, unknown | SR-Q | NA | NA | NA | NA | Yes | No |
| - Adherence to cervical cancer screening guideline^3^ | Never have a smear cervical test  Ever have a smear cervical test, not adherence to guideline  Ever have a smear cervical test, adherence to guideline  Ever have a smear cervical test, >65 years of age  Unknown | SR-Q | NA | NA | NA | NA | Yes | No |
| - Townsend score | Interquartile of Townsend score in the population study: ≤-3.668  (3.668, -2.206]  (-2.206, -0.360]  >0.360 | UK data service | NA | NA | NA | NA | Yes | No |
| - Educational attainment | College or University degree  A levels  AS levels or equivalent  O levels  GCSEs  CSEs or equivalent  Other  None of the above | SR-Q | NA | NA | NA | NA | Yes | No |
| - Occupation | Managers and Senior Officials  Professional Occupations  Associate Professional and Technical Occupations  Administrative and Secretarial Occupations  Skilled Trades Occupations Personal Service Occupations  Sales and Customer Service Occupations  Process, Plant and Machine Operatives  Elementary Occupations  Unknown | SR-Q | NA | NA | NA | NA | Yes | No |
| BMI: Body-mass index, HD: Hospital inpatient databases, ICD: International classification of diseases, NA: Not application, OPCS: OPCS Classification of Interventions and Procedures, PM: physical measurement, SR-I: Self-reported data - Interview with trained nurses, SR-Q: Self-reported data – Questionnaire  ^1^: Include Hospital Episode Statistics Admitted Patient Care (England), Patient Episode Database for Wales Admitted Patient Care (Wales), and General Acute Inpatient and Day Case - Scottish Morbidity Record (Scotland)  ^2^: Following IPAQ guideline (2) | | | | | | | | |
| ^3^: Adherence to cervical cancer screening guideline was defined as having smear test in the last 3 years for women aged less than 50 years and in the last 5 years for women aged from 50-64 years | | | | | | | | |

## Appendix 3: Definition of diabetes

Self-reported, ICD-9 and ICD-10 codes for diabetes

| **Comorbidities** | | **Self-reported data** | **Hospital inpatient databases** | |
| --- | --- | --- | --- | --- |
|  |  |  | **ICD-9** | **ICD-10** |
| Diabetes^1^ | Type 1 diabetes | Algorithm developed by Eastwood et al., 2016 (3) | 25001, 25011 | E10 |
|  | Type 2 diabetes/unknown type of diabetes |  | 250, 2500, 25000, 25009, 2501, 25010, 25019, 2502, 25020, 25021, 25029, 2503, 2504, 2509, 25099 | E11, E12, E13, E14 |

^1^: See “Decision flowchart to identify and classify diabetes” for the final classification

Decision flowchart to identify and classify diabetes


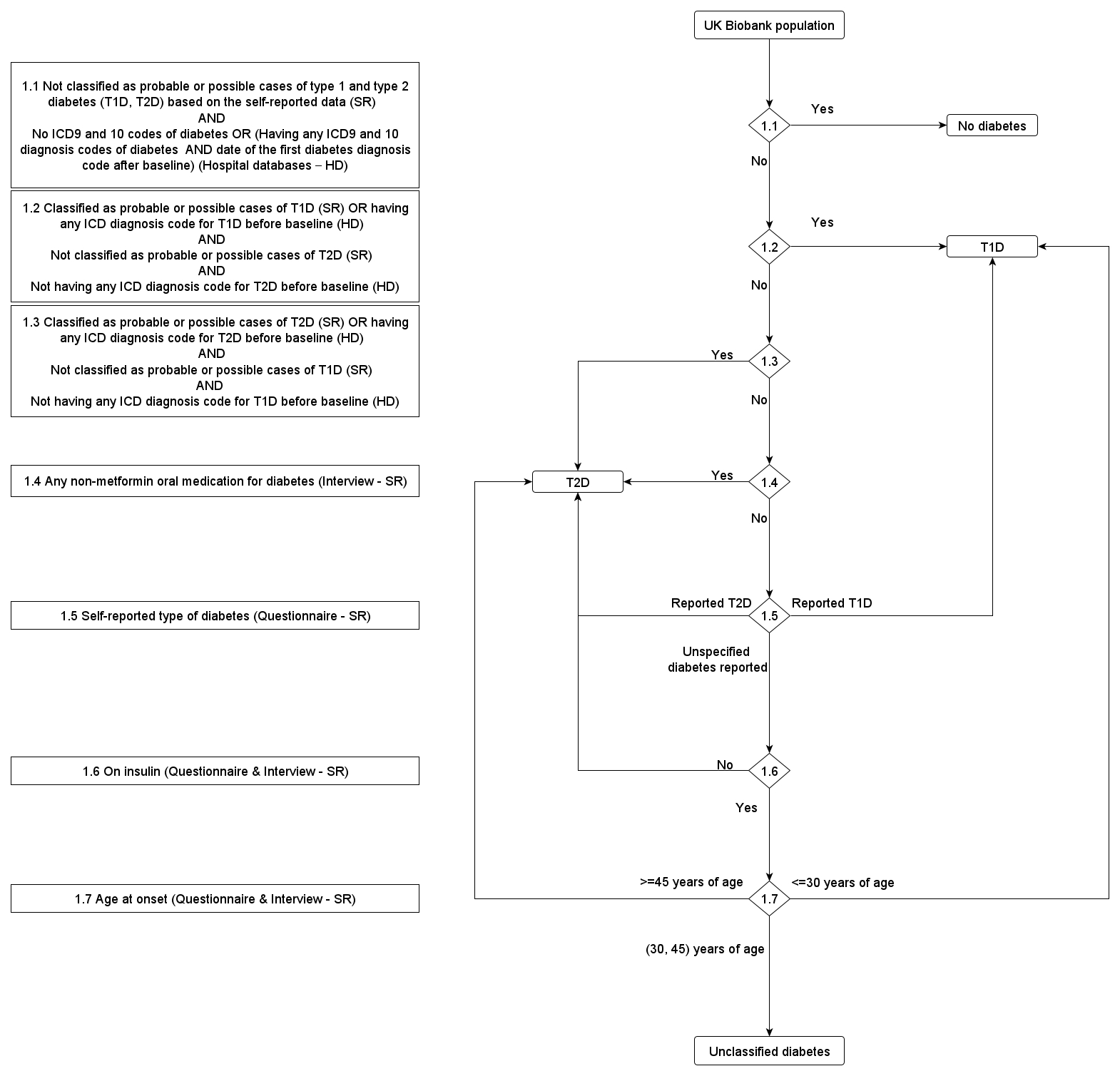


## Appendix 4: List of autoimmune diseases considered in the study

Common autoimmune diseases were identified by including conditions in previous studies (4-6) and hand-searching in the UKB list of self-reported non-cancerous illnesses (Field 20002: <https://biobank.ndph.ox.ac.uk/showcase/field.cgi?id=20002>)

| **No** | **Conditions** | | **Reported code** | **ICD-9** | **ICD-10** | **Note** |
| --- | --- | --- | --- | --- | --- | --- |
| 1 | Type 1 diabetes | Type 1 diabetes | See [appendix 2](#_Appendix_1:_Definition) | See [appendix 2](#_Appendix_1:_Definition) | See [appendix 2](#_Appendix_1:_Definition) |  |
| 1  2 | Inflammatory bowel disease | Inflammatory bowel disease | 1461 | 555, 556 | K50, K51 |  |
|  |  | Crohns disease | 1462 |  |  |  |
|  |  | Ulcerative colitis | 1463 |  |  |  |
| 3 | Primary biliary cirrhosis | Primary biliary cirrhosis | 1506 | 571,6 | K743 |  |
| 4 | Sclerosing cholangitis | Sclerosing cholangitis | 1475 | 576,1 | K830 |  |
| 5 | Glomerulnephritis | Glomerulnephritis | 1609 | 582 | N03 |  |
| 6 | Adrenocortical insufficiency/Addison's disease | Adrenocortical insufficiency/Addison's disease | 1234 | 255,4 | E271 |  |
| 7 | Ankylosing spondylitis | Ankylosing spondylitis | 1313 | 720 | M45 |  |
| 8 | Rheumatoid arthritis | Rheumatoid arthritis | 1464 | 714.0, 714.1, 714.2 | M05, M06 |  |
| 9 | Psoriatic arthropathy | Psoriatic arthropathy | 1477 | 696 | M07 |  |
| 10 | Fibromyalgia | Fibromyalgia | 1542 | 729,1 | M797 |  |
| 11 | Psoriasis | Psoriasis | 1453 | 696.0, 696.1 | L40 |  |
| 12 | Pernicious anaemia | Pernicious anaemia | 1331 | 281.0 | D510 |  |
| 13 | Pemphigoid/pemphigus | Pemphigoid/pemphigus | 1345 | 694.4, 694.5 | L10, L12 |  |
| 14 | Vitiligo | Vitiligo | 1661 | 709,01 | L80 |  |
| 15 | Endometriosis | Endometriosis | 1402 | 617 | N80 |  |
| 16 | Sarcoidosis | Sarcoidosis | 1371 | 135 | D86 |  |
| 17 | Vasculitis | Vasculitis | 1372 | 446.0, 446.4, 466.5, 725, 136.1, 446.21 | M300, M310, M313, M315, M316, M317, M352, M353 |  |
|  |  | Giant cell/temporal arteritis | 1376 |  |  |  |
|  |  | Polymyalgia rheumatica | 1377 |  |  |  |
|  |  | Wegners granulmatosis | 1378 |  |  |  |
|  |  | Microscopic polyarteritis | 1379 |  |  |  |
|  |  | Polyartertis nodosa | 1380 |  |  |  |
|  |  | Behcet’s syndrome | No corresponding code |  |  |  |
|  |  | Goodpasture’s syndrome | No corresponding code |  |  |  |
| 18 | Systemic lupus erythematosis/sle | Systemic lupus erythematosis/sle | 1381 | 710 | M32 |  |
| 19 | Sjogren's syndrome/sicca syndrome | Sjogren's syndrome/sicca syndrome | 1382 | 710,2 | M350 |  |
| 19  20 | Dermatopolymyositis | Dermatopolymyositis | 1383 | 710.3, 710.4 | M33 |  |
|  |  | Dermatomyositis | 1480 |  |  |  |
|  |  | Polymyositis | 1481 |  |  |  |
| 21 | Scleroderma/systemic sclerosis | Scleroderma/systemic sclerosis | 1384 | 710,1 | M34 |  |
| 22 | Raynaud's phenomenon/disease | Raynaud's phenomenon/disease | 1561 | 443.0 | I73.0 |  |
| 23 | Multiple sclerosis | Multiple sclerosis | 1261 | 340 | G35 |  |
| 24 | Malabsorption/coeliac disease | Malabsorption/coeliac disease | 1456 | 579 | K900 |  |
| 25 | Guillain–Barre´ syndrome | Acute infective polyneuritis/guillain-barre syndrome | 1256 | 3570 | G610 |  |
| 26 | Idiopathic fibrosing alveolitis | Fibrosing alveolitis/unspecified alveolitis | 1122 | 51631 | J84112 |  |
| 27 | Myasthenia gravis | Myasthenia gravis | 1260, 1437 | 3580 | G700 |  |
| 28 | Rheumatic fever/heart disease | Rheumatic fever | 1479 | 390, 391, 392 | I00, I01, I02 |  |
| 29 | Chagas disease | Chagas disease | No corresponding code | 086.0, 086.1, 086.2 | B57 |  |
| 30 | Autoimmune hemolytic anemia | Autoimmune hemolytic anemia | No corresponding code | 2830 | D590, D591 |  |
| 31 | Autoimmune thrombocytopenic purpura | Autoimmune thrombocytopenic purpura | No corresponding code | 28731 | D693 |  |
| 32 | Autoimmune hepatitis | Autoimmune hepatitis | No corresponding code | 57142 | K754 |  |
| 33 | Juvenile idiopathic arthritis | Juvenile idiopathic arthritis | No corresponding code | 7143 | M08 |  |
| 34 | Lambert–Eaton myasthenic syndrome | Lambert–Eaton myasthenic syndrome | No corresponding code | 3583 | G7080, G7081 |  |
| 35 | Autoimmune Myocarditis | Autoimmune Myocarditis | No corresponding code | No specific code | No specific code | Not included in the study |
| 36 | Polyendocrine syndromes | Polyendocrine syndromes | No corresponding code | 2581 | E310 |  |
| 37 | Relapsing polychondritis | Relapsing polychondritis | No corresponding code | No specific code | M941 |  |
| 38 | Uveitis | Uveitis | No corresponding code | 3601 | H4413 |  |
| 39 | Alopecia areata | Alopecia areata | No corresponding code | 70400 | L63 |  |

ICD: International classification of diseases

## Appendix 5: Supplementary results

### Supplementary table 1: Baseline characteristics of the study population (n=239,436)

|  | **No thyroid dysfunction reported (n=217,451)** | **Hyperthyroidism (n=3,227)^1^** | | **Hypothyroidism (n=20,762)^1^** | |
| --- | --- | --- | --- | --- | --- |
|  |  |  | **P-value^2^** |  | **P-value^2^** |
| **Alcohol consumption,** n (%) |  |  | <0.001 |  | <0.001 |
| Never | 20,020 (9.2) | 375 (11.6) |  | 2,594 (12.5) |  |
| Once or twice a week or less | 116,055 (53.4) | 1,855 (57.5) |  | 11,857 (57.1) |  |
| Three times a week or more | 80,768 (37.1) | 990 (30.7) |  | 6,260 (30.2) |  |
| Unknown | 608 (0.3) | 7 (0.2) |  | 51 (0.2) |  |
| **Smoking,** n (%) |  |  | <0.001 |  | <0.001 |
| Current smoker | 18,940 (8.7) | 382 (11.8) |  | 1,598 (7.7) |  |
| Former smoker | 65,680 (30.2) | 1,117 (34.6) |  | 7,263 (35.0) |  |
| Never smoker | 131,670 (60.6) | 1,706 (52.9) |  | 11,771 (56.7) |  |
| Unknown | 1,161 (0.5) | 22 (0.7) |  | 130 (0.6) |  |
| **Ethnicity,** n (%) |  |  | 0.005 |  | <0.001 |
| White | 203,937 (93.8) | 3,017 (93.5) |  | 19,726 (95.0) |  |
| Asia | 4,646 (2.1) | 78 (2.4) |  | 515 (2.5) |  |
| Black and Caribbean | 4,072 (1.9) | 80 (2.5) |  | 179 (0.9) |  |
| Other/unknown | 4,796 (2.2) | 52 (1.6) |  | 342 (1.6) |  |
| **Adherence to mammography guideline,** n (%) |  |  | <0.001 |  | <0.001 |
| <50 years of age | 55,489 (25.5) | 594 (18.4) |  | 2,994 (14.4) |  |
| >50 years of age, >3 years ago | 7,102 (3.3) | 123 (3.8) |  | 779 (3.8) |  |
| >50 years of age, in the last 3 years | 142,671 (65.6) | 2,321 (71.9) |  | 15,877 (76.5) |  |
| >50 years of age, never | 7,396 (3.4) | 109 (3.4) |  | 568 (2.7) |  |
| >50 years of age, unknown | 4,793 (2.2) | 80 (2.5) |  | 544 (2.6) |  |
| **Adherence to cervical cancer screening guideline,** n (%) |  |  | <0.001 |  | <0.001 |
| Never have a smear cervical test | 5,049 (2.3) | 69 (2.1) |  | 550 (2.6) |  |
| Ever have a smear cervical test, not adherence to guideline | 23,368 (10.7) | 347 (10.8) |  | 2,493 (12.0) |  |
| Ever have a smear cervical test, adherence to guideline | 138,113 (63.5) | 1,929 (59.8) |  | 11,424 (55.0) |  |
| Ever have a smear cervical test, >65 years of age | 49,892 (22.9) | 873 (27.1) |  | 6,196 (29.8) |  |
| Unknown | 1,029 (0.5) | 9 (0.3) |  | 99 (0.5) |  |
| **Townsend score,** Median (IQR) | -2.2 (-3.6, 0.4) | -2.0 (-3.6, 0.6) | 0.03 | -2.1 (-3.6, 0.6) | <0.001 |
| **Educational attainment,** n (%) |  |  | <0.001 |  | <0.001 |
| College or University degree | 69,155 (31.8) | 885 (27.4) |  | 5,336 (25.7) |  |
| A levels/AS levels or equivalent | 25,924 (11.9) | 365 (11.3) |  | 2,176 (10.5) |  |
| O levels/GCSEs/CSEs or equivalent | 61,669 (28.4) | 911 (28.2) |  | 6,067 (29.2) |  |
| Other | 21,747 (10.0) | 375 (11.6) |  | 2,327 (11.2) |  |
| None of the above | 38,956 (17.9) | 691 (21.4) |  | 4,856 (23.4) |  |
| **Occupation**, n (%) |  |  | <0.001 |  | <0.001 |
| Managers and Senior Officials | 16,542 (7.6) | 220 (6.8) |  | 1,197 (5.8) |  |
| Professional Occupations | 29,405 (13.5) | 374 (11.6) |  | 2,163 (10.4) |  |
| Associate Professional and Technical Occupations | 25,310 (11.6) | 343 (10.6) |  | 2,010 (9.7) |  |
| Administrative and Secretarial Occupations | 31,970 (14.7) | 421 (13.0) |  | 2,916 (14.0) |  |
| Skilled Trades Occupations | 2,234 (1.0) | 31 (1.0) |  | 179 (0.9) |  |
| Personal Service Occupations | 12,818 (5.9) | 170 (5.3) |  | 1,121 (5.4) |  |
| Sales and Customer Service Occupations | 6,781 (3.1) | 88 (2.7) |  | 649 (3.1) |  |
| Process, Plant and Machine Operatives | 1,424 (0.7) | 31 (1.0) |  | 125 (0.6) |  |
| Elementary Occupations | 6,070 (2.8) | 76 (2.4) |  | 597 (2.9) |  |
| Unknown | 84,897 (39.0) | 1,473 (45.6) |  | 9,805 (47.2) |  |

^1^ Women with both hyper- and hypothyroidism reported/recorded (n=2,004) contributed to both columns of hyper- and hypothyroidism

^2^ P-value of t-test, Mann-Whitney U test and χ2 test, where appropriate

### Supplementary table 2: Baseline characteristics of hyperthyroid women (n=3,227)

|  | **Hyperthyroidism without information on treatment (n=1,834)** | **Treated hyperthyroidism (n=1,393)** | **No thyroid dysfunction (n=217,451)** |
| --- | --- | --- | --- |
| **Person-years of follow-up,** median (IQR) | 7.1 (6.4, 7.8) | 7.1 (6.4, 7.9) | 7.1 (6.4, 7.8) |
| **Age at baseline,** Mean (SD) | 56.4 ± 8.1 | 57.8 ± 7.6 | 58.2 ± 7.7 |
| **Age at diagnosis** |  |  |  |
| Women with available information | 1,823 | 1,351 | - |
| Median (IQR) | 46.6 (38.4, 54.6) | 40.5 (29.2, 50.5) | - |
| **Calendar year at diagnosis** |  |  |  |
| Before 1990 | 406 (22.1) | 581 (41.7) | - |
| 1990-2000 | 522 (28.5) | 319 (22.9) | - |
| After 2000 | 895 (48.8) | 452 (32.4) | - |
| Unknown | 11 (0.6) | 41 (2.9) | - |
| **Time interval between diagnosis and baseline** |  |  |  |
| <5 years | 565 (30.8) | 308 (22.1) | - |
| 5-10 years | 446 (24.3) | 189 (13.6) | - |
| >10 years | 812 (44.3) | 854 (61.3) | - |
| Unknown | 11 (0.6) | 42 (3.0) | - |
| **Age at treatment** |  |  |  |
| Women with available information at baseline |  | 1,100 | - |
| At baseline, mean (SD) | - | 39.2 ± 12.6 | - |
| Women with available information at baseline and during the follow-up time | 54 | 1,118 | - |
| At baseline and during the follow-up time, mean (SD) | 50.4 ± 11.0 | 39.5 ± 12.7 | - |
| **Type of treatment at baseline and during the follow-up** |  |  |  |
| Anti-thyroid medications | 0 (0.0) | 270 (19.4) | - |
| RAI | 3 (0) | 383 (27) | - |
| Thyroidectomy | 51 (2.8) | 740 (53.1) | - |
| **Time interval between diagnosis and treatment** |  |  |  |
| Women with available information at baseline |  | 1,089 | - |
| At baseline, median (IQR) | - | 0.2 (0.0, 2.6) | - |
| Women with available information at baseline and during the follow-up time | 54 | 1,106 | - |
| At baseline and during the follow-up time, median (IQR) | 0.0 (0.0, 4.4) | 0.5 (0.0, 2.7) | - |
| **Regular use of beta-blocker at baseline^1^, n (%)** | 4 (0.2) | 3 (0.2) | 244 (0.1) |
| **Regular use of anti-thyroid medications at baseline, n (%)** | 0 (0.0) | 302 (21.7) |  |
| **Regular use of THRT at baseline, n (%)** | 1,010 (55.1) | 905 (65.0) |  |
| **Type of thyroidectomy, n (%)** |  |  |  |
| *At baseline: |  |  |  |
| Total thyroidectomy | - | 59 (8.1) | - |
| Sub-total thyroidectomy | - | 39 (5.4) | - |
| Partial thyroidectomy | - | 32 (4.4) | - |
| Unknown | - | 594 (82.0) | - |
| *At baseline and during the follow-up time |  |  |  |
| Total thyroidectomy | 18 (35.3) | 69 (9.3) | - |
| Sub-total thyroidectomy | 11 (21.6) | 42 (5.7) | - |
| Partial thyroidectomy | 21 (41.2) | 34 (4.6) | - |
| Unknown | 1 (2.0) | 595 (80.4) | - |
| **Menopause status at baseline** |  |  |  |
| Still had periods | 360 (19.6) | 252 (18.1) | 60,047 (27.6) |
| Had menopause before the age of 51 | 1,001 (54.6) | 763 (54.8) | 106,860 (49.1) |
| Had menopause after the age of 51 | 473 (25.8) | 378 (27.1) | 50,544 (23.2) |
| **Age at menopause,** Mean (SD) | 49.1 ± 5.5 | 49.4 ± 5.2 | 49.3 ± 5.1 |
| **Age at menarche,** Mean (SD) | 12.9 ± 1.6 | 13.0 ± 1.7 | 13.0 ± 1.6 |
| **Family history of breast cancer, n (%)** | 174 (9.5) | 135 (9.7) | 22,951 (10.6) |
| **Ever use of menopausal hormone therapy^2^**, n (%) |  |  |  |
| No | 677 (45.9) | 516 (45.2) | 77,660 (49.3) |
| Yes, for less than 5 years | 259 (17.6) | 183 (16) | 27,624 (17.5) |
| Yes, for more than 5 years | 423 (28.7) | 341 (29.9) | 41,604 (26.4) |
| Yes, unknown duration | 111 (7.5) | 95 (8.3) | 9,723 (6.2) |
| Unknown | 4 (0.3) | 6 (0.5) | 793 (0.5) |
| **Ever use of oral contraception,** n (%) |  |  |  |
| No | 39,790 (18.3) | 411 (22.4) | 312 (22.4) |
| Yes, for less than 10 years | 78,959 (36.3) | 703 (38.3) | 513 (36.8) |
| Yes, for more than 10 years | 78,024 (35.9) | 552 (30.1) | 420 (30.2) |
| Yes, unknown duration | 20,339 (9.4) | 164 (8.9) | 144 (10.3) |
| Unknown | 355 (0.2) | 4 (0.2) | 4 (0.3) |
| **Parity and age at first birth, n (%)** |  |  |  |
| No of live birth | 332 (18.1) | 235 (16.9) | 41,026 (18.9) |
| ≥ one chile, <30 years old at birth | 1,209 (65.9) | 925 (66.4) | 135,291 (62.2) |
| ≥ one child, ≥30 years old at birth | 286 (15.6) | 230 (16.5) | 40,071 (18.4) |
| Unknown | 7 (0.4) | 3 (0.2) | 1,063 (0.5) |
| **Levels of physical activity, n (%)** |  |  |  |
| Low | 624 (34.0) | 482 (34.6) | 68,804 (31.6) |
| Moderate | 660 (36.0) | 486 (34.9) | 77,862 (35.8) |
| High | 550 (30.0) | 425 (30.5) | 70,785 (32.6) |
| **Alcohol consumption, n (%)** |  |  |  |
| Never | 227 (12.4) | 148 (10.6) | 20,020 (9.2) |
| Once or twice a week or less | 1,044 (56.9) | 811 (58.2) | 116,055 (53.4) |
| Three times a week or more | 559 (30.5) | 431 (30.9) | 80,768 (37.1) |
| Unknown | 4 (0.2) | 3 (0.2) | 608 (0.3) |
| **Smoking, n (%)** |  |  |  |
| Current smoker | 214 (11.7) | 168 (12.1) | 18,940 (8.7) |
| Former smoker | 610 (33.3) | 507 (36.4) | 65,680 (30.2) |
| Never smoker | 996 (54.3) | 710 (51.0) | 131,670 (60.6) |
| Unknown | 14 (0.8) | 8 (0.6) | 1,161 (0.5) |
| **Adherence to mammography guideline, n (%)** |  |  |  |
| <50 years of age | 340 (18.5) | 254 (18.2) | 55,489 (25.5) |
| >50 years of age, >3 years ago | 72 (3.9) | 51 (3.7) | 7,102 (3.3) |
| >50 years of age, in the last 3 years | 1,302 (71.0) | 1,019 (73.2) | 142,671 (65.6) |
| >50 years of age, never | 68 (3.7) | 41 (2.9) | 7,396 (3.4) |
| >50 years of age, unknown | 52 (2.8) | 28 (2.0) | 4,793 (2.2) |
| **Adherence to cervical cancer screening guideline, n (%)** |  |  |  |
| Never have a smear cervical test | 43 (2.3) | 26 (1.9) | 5,049 (2.3) |
| Ever have a smear cervical test, not adherence to guideline | 211 (11.5) | 136 (9.8) | 23,368 (10.7) |
| Ever have a smear cervical test, adherence to guideline | 1,091 (59.5) | 838 (60.2) | 138,113 (63.5) |
| Ever have a smear cervical test, >65 years of age | 484 (26.4) | 389 (27.9) | 49,892 (22.9) |
| Unknown | 5 (0.3) | 4 (0.3) | 1,029 (0.5) |
| **Townsend score,** Median (IQR) | -1.9 (-3.6, 0.7) | -2.2 (-3.6, 0.3) | -2.2 (-3.6, 0.4) |
| **Educational attainment, n (%)** |  |  |  |
| College or University degree | 526 (28.7) | 359 (25.8) | 69,155 (31.8) |
| A levels/AS levels or equivalent | 212 (11.6) | 153 (11.0) | 25,924 (11.9) |
| O levels/GCSEs/CSEs or equivalent | 517 (28.2) | 394 (28.3) | 61,669 (28.4) |
| Other | 202 (11.0) | 173 (12.4) | 21,747 (10.0) |
| None of the above | 377 (20.6) | 314 (22.5) | 38,956 (17.9) |
| **Occupation, n (%)** |  |  |  |
| Managers and Senior Officials | 126 (6.9) | 94 (6.7) | 16,542 (7.6) |
| Professional Occupations | 233 (12.7) | 141 (10.1) | 29,405 (13.5) |
| Associate Professional and Technical Occupations | 190 (10.4) | 153 (11.0) | 25,310 (11.6) |
| Administrative and Secretarial Occupations | 236 (12.9) | 185 (13.3) | 31,970 (14.7) |
| Skilled Trades Occupations | 17 (0.9) | 14 (1.0) | 2,234 (1.0) |
| Personal Service Occupations | 91 (5.0) | 79 (5.7) | 12,818 (5.9) |
| Sales and Customer Service Occupations | 47 (2.6) | 41 (2.9) | 6,781 (3.1) |
| Process, Plant and Machine Operatives | 18 (1.0) | 13 (0.9) | 1,424 (0.7) |
| Elementary Occupations | 48 (2.6) | 28 (2.0) | 6,070 (2.8) |
| Unknown | 828 (45.1) | 645 (46.3) | 84,897 (39.0) |
| **Corpulence, n (%)** |  |  |  |
| Obesity/Overweight, BMI $\geq$ 25 kg/m² | 1,125 (61.3) | 849 (60.9) | 128,257 (59.0) |
| Normal weight, BMI  18.5-25 kg/m^2^ | 698 (38.1) | 541 (38.8) | 88,047 (40.5) |
| Unknown | 11 (0.6) | 3 (0.2) | 1,147 (0.5) |
| **Comorbidities, n (%)** |  |  |  |
| Type 2 diabetes, n (%) | 101 (5.5) | 65 (4.7) | 6,535 (3.0) |
| Hypertension, n (%) | 572 (31.2) | 434 (31.2) | 49,855 (22.9) |
| Depression, n (%) | 162 (8.8) | 101 (7.3) | 15,146 (7.0) |
| Autoimmune diseases, n (%) | 270 (14.7) | 180 (12.9) | 20,266 (9.3) |

BMI: Body-mass index, RAI: Radioactive iodine therapy THRT: Thyroid hormone replacement therapy

^1^ Atenolol and propranolol

^2^ Postmenopausal women only

### Supplementary table 3: Hazard ratios of breast cancer incidence associated with hyperthyroidism diagnosis versus no thyroid dysfunction at baseline, stratified according to treatment status

| **Characteristics** | **Breast cancer (n)** | **Person-years** | **HR** | **95% CI** |
| --- | --- | --- | --- | --- |
| **No thyroid dysfunction (reference)** | 4,854 | 1,518,670.0 | **1** | **—** |
| **Time since hyperthyroidism diagnosis** |  |  |  |  |
| Hyperthyroidism without information on treatment |  |  |  |  |
| - ≤5 years ago | 1 | 1,400.9 | 0.3 | 0.04, 2.14 |
| - 5-10 years ago | 14 | 3,379.8 | 1.3 | 0.77, 2.19 |
| - > 10 years ago | 20 | 7,958.5 | 0.74 | 0.48, 1.15 |
| - Unknown time at diagnosis | 0 | 77.0 | - | - |
| Treated hyperthyroidism |  |  |  |  |
| - ≤5 years ago | 3 | 932.8 | 1.26 | 0.41, 3.91 |
| - 5-10 years ago | 11 | 1,567.6 | **2.16** | **1.20, 3.91** |
| - > 10 years ago | 29 | 6,929.1 | 1.24 | 0.86, 1.79 |
| - Unknown time at diagnosis | 1 | 275.0 | 1.13 | 0.16, 8.05 |
| **Age at hyperthyroidism diagnosis** |  |  |  |  |
| Hyperthyroidism without information on treatment |  |  |  |  |
| - ≤40 years of age | 6 | 7,310.2 | 0.52 | 0.24, 1.17 |
| - 40-60 years of age | 22 | 7,337.1 | 0.93 | 0.61, 1.41 |
| - > 60 years of age | 6 | 1,373.7 | 1.17 | 0.52, 2.6 |
| - Unknown age at diagnosis | 0 | 77.0 | - | - |
| Treated hyperthyroidism |  |  |  |  |
| - ≤40 years of age | 14 | 4,726.1 | 0.92 | 0.54, 1.56 |
| - 40-60 years of age | 23 | 4,387.9 | **1.61** | **1.07, 2.42** |
| - > 60 years of age | 7 | 633.5 | **2.97** | **1.41, 6.24** |
| - Unknown age at diagnosis | 1 | 275.0 | 1.15 | 0.16, 8.2 |

HR = Hazard Ratio, CI = Confidence Interval

HRs are adjusted for age at baseline (continuous), family history of breast cancer (yes/no), parity and number of live birth (No live birth/$\geq$ one child, <30 years old at birth/$\geq$ one child, $\geq$30 years old at birth/Unknown), menopausal status (premenopause/postmenopause before the age of 51/postmenopause after the age of 51), physical activity (Low/Moderate/High)

### Supplementary table 4: Hyperthyroidism sensitivity analyses

| **Sensitivity analyses** | **Hyperthyroidism status** | **Breast cancer (n)** | **Person-years** | **aHR** | **95% CI** |
| --- | --- | --- | --- | --- | --- |
| No other thyroid problems at baseline | No thyroid dysfunction (reference) | 4821 | 1511069 | 1 |  |
|  | Hyperthyroidism | 77 | 21438 | 1.10 | 0.88, 1.38 |
|  | - Hyperthyroidism without information on treatment | 35 | 12376 | 0.87 | 0.62, 1.21 |
|  | - Treated hyperthyroidism | 42 | 9063 | **1.42** | **1.05, 1.93** |
|  | Age at hyperthyroidism diagnosis |  |  |  |  |
|  | - Before 40 years old | 20 | 8156.8 | 0.78 | 0.50, 1.20 |
|  | - Between 40-60 years old | 43 | 11076.0 | 1.2 | 0.89, 1.62 |
|  | - After 60 years old | 13 | 1907.7 | **1.82** | **1.06, 3.15** |
|  | - Unknown age at diagnosis | 1 | 297.8 | 1.1 | 0.15, 7.80 |
|  | Time since hyperthyroidism diagnosis |  |  |  |  |
|  | - Less than 5 years ago | 4 | 2240.7 | 0.73 | 0.27, 1.95 |
|  | - Between 5-10 years ago | 25 | 4660.6 | **1.67** | **1.13, 2.47** |
|  | - More than 10 years ago | 47 | 14239.2 | 0.98 | 0.73, 1.30 |
|  | - Unknown time at diagnosis | 1 | 297.8 | 1.11 | 0.16, 7.85 |
| Order of hyper- and hypothyroidism occurrence | No thyroid dysfunction (reference) | 4854 | 1518670 | 1 | — |
|  | Only hyperthyroidism | 30 | 8501.5 | 1.08 | 0.76, 1.55 |
|  | Hyperthyroidism before hypothyroidism | 21 | 6645.8 | 0.97 | 0.63, 1.49 |
|  | Hyperthyroidism after hypothyroidism | 4 | 1488.9 | 0.83 | 0.31, 2.21 |
|  | Both hyper- and hypothyroidism, unknown time sequence | 24 | 5884.4 | 1.25 | 0.84, 1.87 |
| Exclusion of hypo- before hyperthyroidism and of thyroid dysfunction with unknown sequential order of occurrence | No thyroid dysfunction (reference) | 4854 | 1518670.0 | 1 |  |
|  | Hyperthyroidism | 51 | 15147.3 | 1.03 | 0.78, 1.36 |
|  | - Hyperthyroidism without information on treatment | 20 | 8635.3 | 0.71 | 0.46, 1.10 |
|  | - Treated hyperthyroidism | 31 | 6511.9 | **1.46** | **1.03, 2.08** |
|  | Age at hyperthyroidism diagnosis |  |  |  |  |
|  | - Before 40 years old | 12 | 5908.5 | 0.64 | 0.36, 1.13 |
|  | - Between 40-60 years old | 31 | 7907.1 | 1.21 | 0.85, 1.72 |
|  | - After 60 years old | 8 | 1205.9 | 1.78 | 0.89, 3.57 |
|  | - Unknown age at diagnosis | 0 | 125.7 | 0.64 | 0.36, 1.13 |
|  | Time since hyperthyroidism diagnosis |  |  |  |  |
|  | - Less than 5 years ago | 3 | 1549.2 | 0.79 | 0.25, 2.45 |
|  | - Between 5-10 years ago | 16 | 3249.9 | 1.54 | 0.94, 2.52 |
|  | - More than 10 years ago | 32 | 10222.4 | 0.92 | 0.65, 1.30 |
|  | - Unknown time at diagnosis | 0 | 125.7 | - | - |
| Thyroid dysfunction identified in hospital inpatient databases | No thyroid dysfunction (reference) | 4854 | 1518780 | 1 |  |
|  | Hyperthyroidism | 17 | 5041 | 1.06 | 0.66, 1.71 |
|  | - Hyperthyroidism without information on treatment | 7 | 3347 | 0.65 | 0.31, 1.37 |
|  | - Treated hyperthyroidism | 10 | 1694 | 1.85 | 0.99, 3.44 |
|  | Age at hyperthyroidism diagnosis |  |  |  |  |
|  | - Before 40 years old | 2 | 1042.3 | 0.67 | 0.17, 2.70 |
|  | - Between 40-60 years old | 10 | 3122.9 | 1.03 | 0.55, 1.91 |
|  | - After 60 years old | 5 | 856.8 | 1.57 | 0.65, 3.79 |
|  | - Unknown age at diagnosis | 0 | 18.6 | - | - |
|  | Time since hyperthyroidism diagnosis |  |  |  |  |
|  | - Less than 5 years ago | 1 | 759.3 | 0.55 | 0.08, 3.90 |
|  | - Between 5-10 years ago | 10 | 1742.5 | 1.8 | 0.97, 3.34 |
|  | - More than 10 years ago | 6 | 2520.1 | 0.71 | 0.32, 1.58 |
|  | - Unknown time at diagnosis | 0 | 18.6 | - | - |
| Thyroid dysfunction identified in self-reported data | No thyroid dysfunction (reference) | 4854 | 1518780 | 1 |  |
|  | Hyperthyroidism | 72 | 19873 | 1.11 | 0.88, 1.40 |
|  | - Hyperthyroidism without information on treatment | 29 | 10474 | 0.85 | 0.59, 1.22 |
|  | - Treated hyperthyroidism | 43 | 9399 | **1.40** | **1.03, 1.88** |
|  | Age at hyperthyroidism diagnosis |  |  |  |  |
|  | - Before 40 years old | 19 | 8118.8 | 0.74 | 0.47, 1.15 |
|  | - Between 40-60 years old | 42 | 10055.5 | 1.28 | 0.94, 1.73 |
|  | - After 60 years old | 10 | 1365.2 | **1.95** | **1.05, 3.62** |
|  | - Unknown age at diagnosis | 1 | 333.4 | 0.98 | 0.14, 6.94 |
|  | Time since hyperthyroidism diagnosis |  |  |  |  |
|  | - Less than 5 years ago | 3 | 1805.9 | 0.68 | 0.22, 2.11 |
|  | - Between 5-10 years ago | 22 | 3876.4 | **1.78** | **1.17, 2.71** |
|  | - More than 10 years ago | 46 | 13857.1 | 0.98 | 0.73, 1.31 |
|  | - Unknown time at diagnosis | 1 | 333.4 | 0.98 | 0.14, 6.93 |
| Invasive breast tumor only | No thyroid dysfunction (reference) | 4050 | 1518780 | 1 |  |
|  | Hyperthyroidism | 69 | 22521 | 1.12 | 0.89, 1.43 |
|  | - Hyperthyroidism without information on treatment | 34 | 12816 | 0.98 | 0.70, 1.37 |
|  | - Treated hyperthyroidism | 35 | 9704 | 1.32 | 0.94, 1.83 |
|  | Age at hyperthyroidism diagnosis |  |  |  |  |
|  | - Before 40 years old | 18 | 8436.4 | 0.81 | 0.51, 1.29 |
|  | - Between 40-60 years old | 38 | 11724.9 | 1.19 | 0.87, 1.64 |
|  | - After 60 years old | 13 | 2007.2 | **2.04** | **1.18, 3.53** |
|  | - Unknown age at diagnosis | 0 | 352.0 | - | - |
|  | Time since hyperthyroidism diagnosis |  |  |  |  |
|  | - Less than 5 years ago | 4 | 2333.6 | 0.84 | 0.31, 2.23 |
|  | - Between 5-10 years ago | 23 | 4947.4 | **1.73** | **1.15, 2.61** |
|  | - More than 10 years ago | 42 | 14887.6 | 1 | 0.73, 1.35 |
|  | - Unknown time at diagnosis | 0 | 352.0 | - | - |
| Updated thyroid dysfunction during the follow-up time | No thyroid dysfunction (reference) | 4821 | 1512089 | 1 |  |
|  | Hyperthyroidism | 87 | 24540 | 1.07 | 0.87, 1.33 |
|  | - Hyperthyroidism without information on treatment | 41 | 14219 | 0.87 | 0.64, 1.18 |
|  | - Treated hyperthyroidism | 46 | 10321 | **1.36** | **1.01, 1.81** |
|  | Age at hyperthyroidism diagnosis |  |  |  |  |
|  | - Before 40 years old | 20 | 8436.4 | 0.75 | 0.48, 1.17 |
|  | - Between 40-60 years old | 45 | 11724.9 | 1.18 | 0.88, 1.59 |
|  | - After 60 years old | 13 | 2007.2 | **1.73** | **1.00, 2.99** |
|  | - Unknown age at diagnosis | 1 | 352.0 | 0.91 | 0.13, 6.49 |
|  | Time since hyperthyroidism diagnosis |  |  |  |  |
|  | - Less than 5 years ago | 12 | 4206.7 | 0.93 | 0.53, 1.63 |
|  | - Between 5-10 years ago | 25 | 5094.0 | **1.51** | **1.02, 2.24** |
|  | - More than 10 years ago | 49 | 14887.6 | 0.97 | 0.73, 1.28 |
|  | - Unknown time at diagnosis | 1 | 352.0 | 0.91 | 0.13, 6.49 |
| Complete-case analysis^1^ | No thyroid dysfunction (reference) | 4501 | 1411609 | 1 |  |
|  | Hyperthyroidism | 73 | 21022 | 1.07 | 0.85, 1.35 |
|  | - Hyperthyroidism without information on treatment | 31 | 11940 | 0.80 | 0.56, 1.14 |
|  | - Treated hyperthyroidism | 42 | 9082 | **1.42** | **1.05, 1.92** |
|  | Age at hyperthyroidism diagnosis |  |  |  |  |
|  | - Before 40 years old | 19 | 8099.1 | 0.74 | 0.47, 1.16 |
|  | - Between 40-60 years old | 40 | 11292.3 | 1.09 | 0.80, 1.48 |
|  | - After 60 years old | 13 | 1950.6 | **1.8** | **1.04, 3.10** |
|  | - Unknown age at diagnosis | 1 | 344.4 | 0.95 | 0.13, 6.71 |
|  | Time since hyperthyroidism diagnosis |  |  |  |  |
|  | - Less than 5 years ago | 4 | 2333.6 | 0.7 | 0.26, 1.87 |
|  | - Between 5-10 years ago | 25 | 4947.4 | **1.58** | **1.06, 2.33** |
|  | - More than 10 years ago | 49 | 14887.6 | 0.97 | 0.73, 1.29 |
|  | - Unknown time at diagnosis | 1 | 352.0 | 0.92 | 0.13, 6.51 |
| HR = Hazard Ratio, CI = Confidence Interval  HRs are adjusted for age at baseline (continuous), family history of breast cancer (yes/no), parity and number of live birth (No live birth/$\geq$ one child, <30 years old at birth/$\geq$ one child, $\geq$30 years old at birth/Unknown), menopausal status (premenopause/postmenopause before the age of 51/postmenopause after the age of 51), physical activity (Low/Moderate/High)  ^1^ Exclude women with missing data in covariates adjusted in Cox proportional models: menopausal status, family history of breast cancer, parity and number of live birth, physical activity | | | | | |

### Supplementary table 5: Hypothyroidism sensitivity analyses

| **Sensitivity analyses** | **Hypothyroidism status** | **Breast cancer (n)** | **Person-years** | **aHR** | **95% CI** |
| --- | --- | --- | --- | --- | --- |
| No other thyroid problems at baseline | No thyroid dysfunction (reference) | 4821 | 1511069 | 1 |  |
|  | Hypothyroidism | 416 | 136506 | 0.93 | 0.84, 1.02 |
|  | Age at hypothyroidism diagnosis |  |  |  |  |
|  | - Before 40 years old | 49 | 23131.6 | **0.72** | **0.54, 0.95** |
|  | - Between 40-60 years old | 264 | 85705.8 | 0.94 | 0.83, 1.06 |
|  | - After 60 years old | 63 | 16115.7 | 1.04 | 0.81, 1.33 |
|  | - Unknown age at diagnosis | 40 | 11546.3 | 1.04 | 0.76, 1.42 |
|  | Time since hyperthyroidism diagnosis |  |  |  |  |
|  | - Less than 5 years ago | 31 | 14623.5 | 0.86 | 0.61, 1.23 |
|  | - Between 5-10 years ago | 125 | 35930.8 | 1.07 | 0.89, 1.28 |
|  | - More than 10 years ago | 220 | 74398.8 | **0.85** | **0.74, 0.97** |
|  | - Unknown time at diagnosis | 40 | 11546.3 | 1.08 | 0.79, 1.47 |
| Order of hyper- and hypothyroidism occurrence | No thyroid dysfunction (reference) | 4854 | 1518670 | 1 | — |
|  | Only hypothyroidism | 393 | 130194.0 | 0.91 | 0.82, 1.01 |
|  | Hyperthyroidism before hypothyroidism | 21 | 6645.8 | 0.97 | 0.63, 1.48 |
|  | Hyperthyroidism after hypothyroidism | 4 | 1488.9 | 0.83 | 0.31, 2.21 |
|  | Both hyper- and hypothyroidism, unknown time sequence | 24 | 5884.4 | 1.24 | 0.83, 1.86 |
| Exclusion of hypo- before hyperthyroidism and of thyroid dysfunction with unknown sequential order of occurrence | No thyroid dysfunction (reference) | 4854 | 1518670.0 | 1 |  |
|  | Hypothyroidism | 414 | 136839.7 | 0.92 | 0.83, 1.02 |
|  | Age at hypothyroidism diagnosis |  |  |  |  |
|  | - Before 40 years old | 50 | 23144.8 | **0.73** | **0.55, 0.96** |
|  | - Between 40-60 years old | 266 | 86915.5 | 0.93 | 0.82, 1.06 |
|  | - After 60 years old | 70 | 16570.9 | 1.12 | 0.88, 1.42 |
|  | - Unknown age at diagnosis | 28 | 10208.6 | 0.82 | 0.57, 1.19 |
|  | Time since hyperthyroidism diagnosis |  |  |  |  |
|  | - Less than 5 years ago | 33 | 15049.3 | 0.89 | 0.63, 1.25 |
|  | - Between 5-10 years ago | 130 | 36707.0 | 1.08 | 0.91, 1.29 |
|  | - More than 10 years ago | 223 | 74874.8 | **0.85** | **0.74, 0.97** |
|  | - Unknown time at diagnosis | 28 | 10208.6 | 0.84 | 0.58, 1.22 |
| Thyroid dysfunction identified in hospital inpatient databases | No thyroid dysfunction (reference) | 4854 | 1518780 | 1 |  |
|  | Hypothyroidism | 94 | 33024 | 0.86 | 0.70, 1.05 |
|  | Age at hypothyroidism diagnosis |  |  |  |  |
|  | - Before 40 years old | 13 | 6696.8 | 0.65 | 0.38, 1.12 |
|  | - Between 40-60 years old | 65 | 22162.2 | 0.88 | 0.69, 1.12 |
|  | - After 60 years old | 16 | 4164.9 | 1.02 | 0.62, 1.66 |
|  | - Unknown age at diagnosis | 0 | 0 |  |  |
|  | Time since hyperthyroidism diagnosis |  |  |  |  |
|  | - Less than 5 years ago | 5 | 2659.3 | 0.74 | 0.31, 1.79 |
|  | - Between 5-10 years ago | 28 | 8141.9 | 1.06 | 0.73, 1.54 |
|  | - More than 10 years ago | 61 | 22222.8 | **0.78** | **0.60, 1.00** |
|  | - Unknown time at diagnosis | 0 | 0 | 0.74 | 0.31, 1.79 |
| Thyroid dysfunction identified in self-report data | No thyroid dysfunction (reference) | 4854 | 1518780 | 1 |  |
|  | Hypothyroidism | 435 | 142184 | 0.93 | 0.84, 1.02 |
|  | Age at hypothyroidism diagnosis |  |  |  |  |
|  | - Before 40 years old | 50 | 23507.6 | **0.72** | **0.54, 0.95** |
|  | - Between 40-60 years old | 268 | 86684.1 | 0.94 | 0.83, 1.06 |
|  | - After 60 years old | 66 | 16068.7 | 1.09 | 0.85, 1.39 |
|  | - Unknown age at diagnosis | 51 | 15923.6 | 0.96 | 0.73, 1.27 |
|  | Time since hyperthyroidism diagnosis |  |  |  |  |
|  | - Less than 5 years ago | 33 | 14625.8 | 0.92 | 0.65, 1.29 |
|  | - Between 5-10 years ago | 126 | 36058.3 | 1.07 | 0.90, 1.28 |
|  | - More than 10 years ago | 225 | 75576.3 | **0.85** | **0.74, 0.97** |
|  | - Unknown time at diagnosis | 51 | 15923.6 | 0.99 | 0.75, 1.30 |
| Invasive breast tumor only | No thyroid dysfunction (reference) | 4050 | 1518780 | 1 |  |
|  | Hypothyroidism | 374 | 144219 | 0.94 | 0.84, 1.04 |
|  | Age at hypothyroidism diagnosis |  |  |  |  |
|  | - Before 40 years old | 40 | 23615.7 | **0.69** | **0.51, 0.94** |
|  | - Between 40-60 years old | 235 | 87945.4 | 0.97 | 0.85, 1.11 |
|  | - After 60 years old | 62 | 16728.3 | 1.16 | 0.90, 1.49 |
|  | - Unknown age at diagnosis | 37 | 15923.6 | 0.83 | 0.60, 1.15 |
|  | Time since hyperthyroidism diagnosis |  |  |  |  |
|  | - Less than 5 years ago | 30 | 15154.9 | 0.95 | 0.66, 1.36 |
|  | - Between 5-10 years ago | 112 | 37024.4 | 1.1 | 0.91, 1.33 |
|  | - More than 10 years ago | 195 | 76110.1 | 0.87 | 0.75, 1.01 |
|  | - Unknown time at diagnosis | 37 | 15923.6 | 0.85 | 0.62, 1.18 |
| Updated thyroid dysfunction during the follow-up time | No thyroid dysfunction (reference) | 4821 | 1512089 | 1 |  |
|  | Hypothyroidism | 471 | 150071 | 0.94 | 0.86, 1.04 |
|  | Age at hypothyroidism diagnosis |  |  |  |  |
|  | - Before 40 years old | 50 | 23615.7 | **0.71** | **0.54, 0.94** |
|  | - Between 40-60 years old | 284 | 90250.0 | 0.95 | 0.85, 1.07 |
|  | - After 60 years old | 86 | 20281.6 | 1.09 | 0.88, 1.36 |
|  | - Unknown age at diagnosis | 51 | 15923.6 | 0.99 | 0.75, 1.30 |
|  | Time since hyperthyroidism diagnosis |  |  |  |  |
|  | - Less than 5 years ago | 62 | 20751.8 | 1.05 | 0.81, 1.34 |
|  | - Between 5-10 years ago | 132 | 37285.4 | 1.08 | 0.91, 1.29 |
|  | - More than 10 years ago | 226 | 76110.1 | **0.85** | **0.74, 0.97** |
|  | - Unknown time at diagnosis | 51 | 15923.6 | 0.99 | 0.75, 1.30 |
| Complete-case analysis^1^ | No thyroid dysfunction (reference) | 4501 | 1411609 | 1 |  |
|  | Hypothyroidism | 416 | 133900 | 0.95 | 0.86, 1.05 |
|  | Age at hypothyroidism diagnosis |  |  |  |  |
|  | - Before 40 years old | 50 | 22122.4 | **0.76** | **0.57, 1.00** |
|  | - Between 40-60 years old | 260 | 85399.8 | 0.93 | 0.82, 1.05 |
|  | - After 60 years old | 70 | 16419.5 | 1.13 | 0.89, 1.44 |
|  | - Unknown age at diagnosis | 51 | 15334.7 | 1 | 0.76, 1.32 |
|  | Time since hyperthyroidism diagnosis |  |  |  |  |
|  | - Less than 5 years ago | 34 | 15154.9 | 0.91 | 0.65, 1.27 |
|  | - Between 5-10 years ago | 131 | 37024.4 | 1.08 | 0.91, 1.29 |
|  | - More than 10 years ago | 226 | 76110.1 | **0.85** | **0.74, 0.97** |
|  | - Unknown time at diagnosis | 51 | 15923.6 | 0.99 | 0.75, 1.30 |
| HR = Hazard Ratio, CI = Confidence Interval  HRs are adjusted for age at baseline (continuous), family history of breast cancer (yes/no), parity and number of live birth (No live birth/$\geq$ one child, <30 years old at birth/$\geq$ one child, $\geq$30 years old at birth/Unknown), menopausal status (premenopause/postmenopause before the age of 51/postmenopause after the age of 51), physical activity (Low/Moderate/High)  ^1^ Exclude women with missing data in covariates adjusted in Cox proportional models: menopausal status, family history of breast cancer, parity and number of live birth, physical activity | | | | | |

### Supplementary table 6: Results from Cox proportional hazard models for death and other cancer incidence as competing risks

| **Characteristics** |  | **Hyperthyroidism** | | |  | **Hypothyroidism** | | |
| --- | --- | --- | --- | --- | --- | --- | --- | --- |
|  | **Death/other cancer incidence (n)** | **aHR** | **95% CI** | **p-value** | **Death/other cancer incidence (n)** | **aHR** | **95% CI** | **p-value** |
| No thyroid dysfunction | 8,339 | — | — |  | 8,339 | — | — |  |
| Thyroid dysfunction | 164 | 1.22 | 1.04, 1.42 | 0.012 | 932 | 1.03 | 0.96, 1.10 | 0.475 |

HRs are adjusted for age at baseline (continuous), family history of breast cancer (yes/no), parity and number of live birth (No live birth/$\geq$ one child, <30 years old at birth/$\geq$ one child, $\geq$30 years old at birth/Unknown), menopausal status (premenopause/postmenopause before the age of 51/postmenopause after the age of 51), physical activity (Low/Moderate/High)

### Supplementary figure 1: Cumulative incidence function of breast cancer accounting for death and other cancer incidence as competing risks


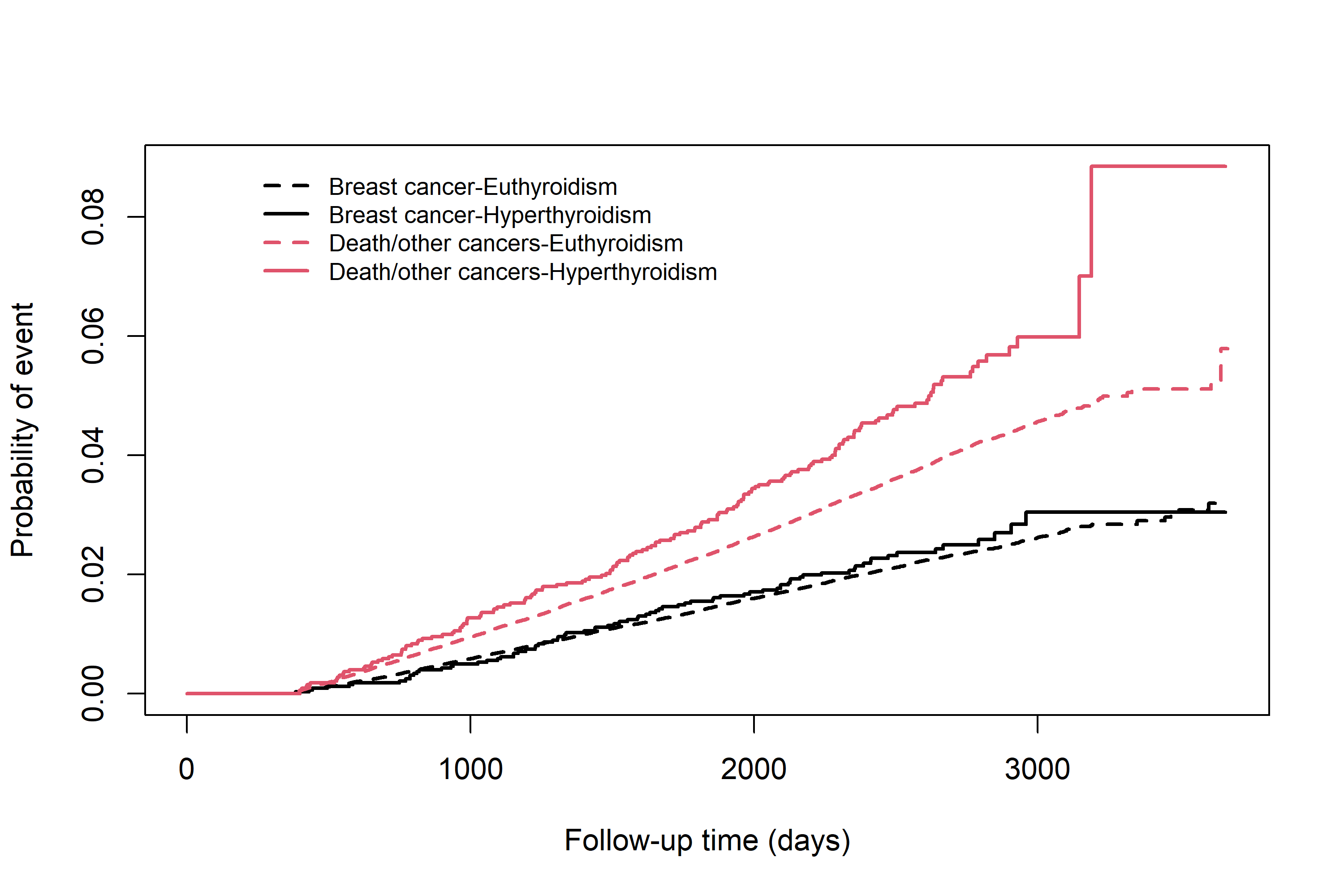

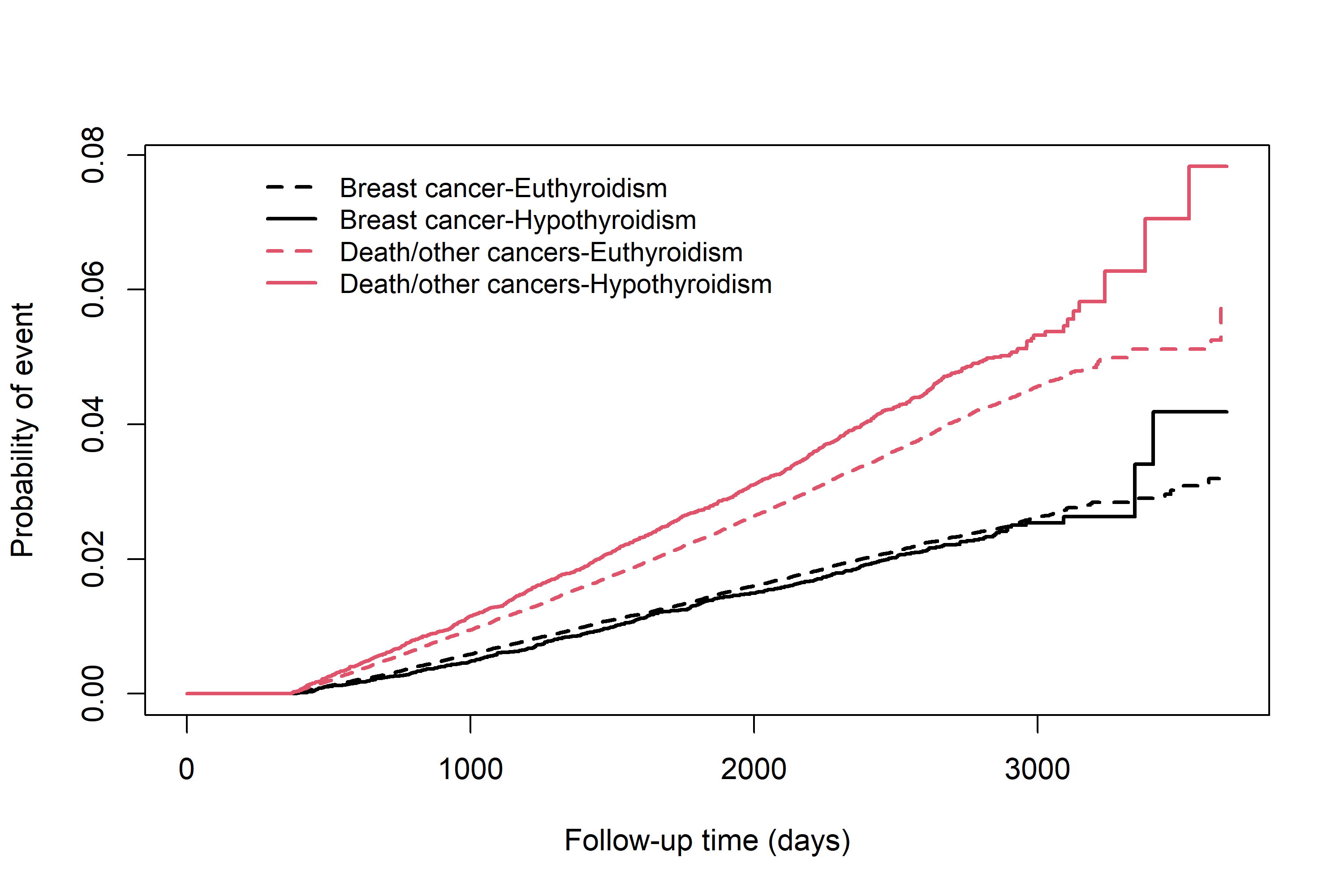


### Supplementary figure 2: Evaluation of effect modification of comorbidities, and breast cancer risk factors in the association between thyroid dysfunction and breast cancer risk.


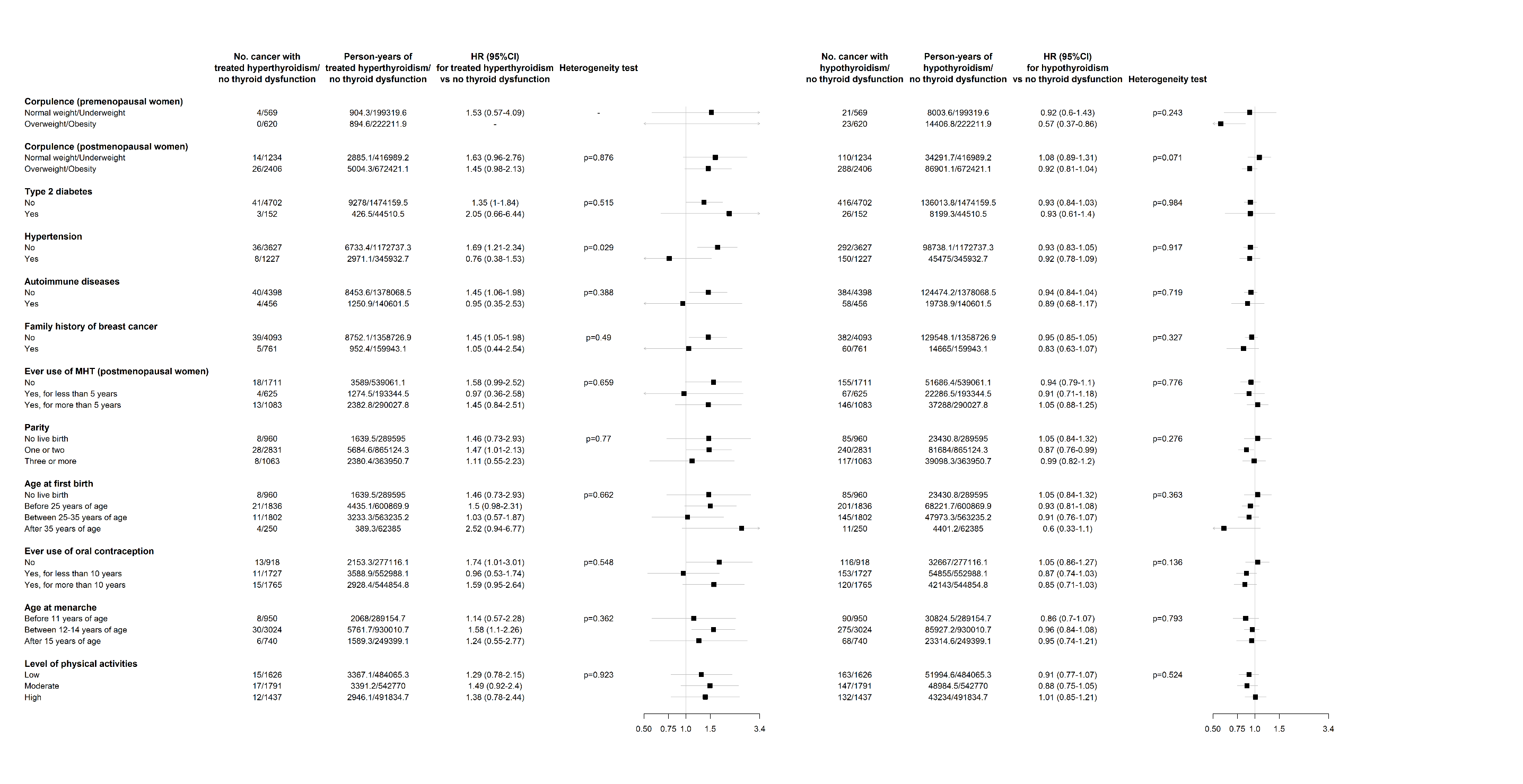


Results for “Unknown” category were not shown. HR: Hazard ratio, CI: Confidence interval

# References

1. Obesity: preventing and managing the global epidemic. Report of a WHO consultation. World Health Organization technical report series. 2000;894:i-xii, 1-253.

2. Fan M, Lyu J, He P. Guidelines for data processing and analysis of the International Physical Activity Questionnaire (IPAQ).2005. URL: <<http://www.IPAQ.ki.se>. Zhonghua liu xing bing xue za zhi = Zhonghua liuxingbingxue zazhi. 2014;35:961-4.

3. Eastwood SV, Mathur R, Atkinson M, Brophy S, Sudlow C, Flaig R, et al. Algorithms for the Capture and Adjudication of Prevalent and Incident Diabetes in UK Biobank. PLoS One. 2016;11(9):e0162388-e.

4. Schairer C, Pfeiffer RM, Gadalla SM. Autoimmune diseases and breast cancer risk by tumor hormone-receptor status among elderly women. International journal of cancer. 2018;142(6):1202-8.

5. Thomas SL, Griffiths C, Smeeth L, Rooney C, Hall AJ. Burden of mortality associated with autoimmune diseases among females in the United Kingdom. American journal of public health. 2010;100(11):2279-87.

6. Eaton WW, Pedersen MG, Atladóttir HO, Gregory PE, Rose NR, Mortensen PB. The prevalence of 30 ICD-10 autoimmune diseases in Denmark. Immunologic research. 2010;47(1-3):228-31.
